# Multifunctional nanoparticles co-loaded with Adriamycin and MDR-targeting siRNAs for treatment of chemotherapy-resistant esophageal cancer

**DOI:** 10.1186/s12951-022-01377-x

**Published:** 2022-03-28

**Authors:** Xiangyang Zhang, Min Wang, Junyi Feng, Bin Qin, Chenglin Zhang, Chengshen Zhu, Wentao Liu, Yaohe Wang, Wei Liu, Lei Huang, Shuangshuang Lu, Zhimin Wang

**Affiliations:** 1 Sino-British Research Centre for Molecular Oncology, National Centre for International Research in Cell and Gene Therapy, School of Basic Medical Sciences, Academy of Medical Sciences, Zhengzhou University, Zhengzhou, Henan People’s Republic of China; 2 School of Material Science and Engineering, Zhengzhou University, Zhengzhou, Henan People’s Republic of China; 3 Children’s Hospital Affiliated to Zhengzhou University, Zhengzhou, Henan People’s Republic of China; 4 Inflammations Immunity Research Theme, Translational and Clinical Research Institute, FMS, Newcastle University, Newcastle Upon Tyne, NE1 7RU UK

**Keywords:** Tumor targeting, Chemotherapy, siRNA, Multidrug resistance, Esophageal cancer

## Abstract

The development of multidrug resistance (MDR) during cancer chemotherapy is a major challenge in current cancer treatment strategies. Numerous molecular mechanisms, including increased drug efflux, evasion of drug-induced apoptosis, and activation of DNA repair mechanisms, can drive chemotherapy resistance. Here we have identified the major vault protein (MVP) and the B-cell lymphoma-2 (BCL2) gene as two potential factors driving MDR in esophageal squamous cell carcinoma (ESCC). We have designed a novel and versatile self-assembling nanoparticle (NP) platform on a multifunctional carboxymethyl chitosan base to simultaneously deliver Adriamycin, and siRNAs targeting MVP and BCL2 (CEAMB NPs), thus reducing drug efflux and promoting apoptosis of esophageal cancer cells. To achieve effective delivery to tumor tissues and inhibit tumor growth in vivo, carboxymethyl chitosan was engineered to contain multiple histidines for enhanced cytosol delivery, cholesterol for improved self-assembly, and epidermal growth factor receptor (EGFR) antibodies to target cancer cells. Our results indicate that these nanoparticles are efficiently synthesized with the desired chemical composition to self-assemble into cargo-containing NPs. Furthermore, we have shown that the synthesized NPs will successfully inhibit cancer cells growth and tumor development when delivered to cultured ESCC cells or to in vivo mouse xenograft models. Our engineered NPs offer a potential novel platform in treating various types of chemotherapy-resistant tumors.

**Graphical Abstract:**

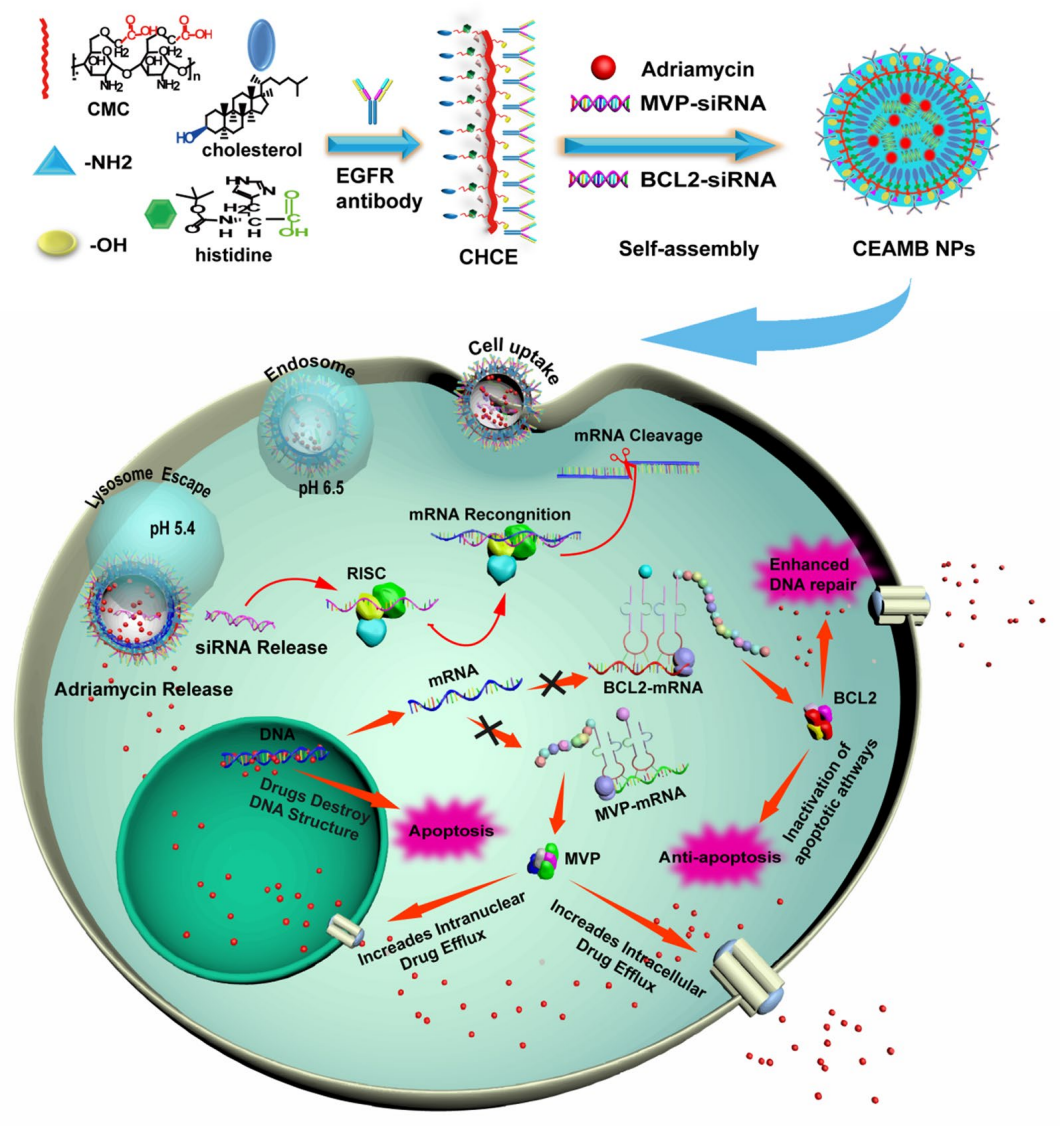

**Supplementary Information:**

The online version contains supplementary material available at 10.1186/s12951-022-01377-x.

## Introduction

Chemotherapies (CT) are first-line cancer treatments. These therapies induce DNA damage and activate a complex cell-signaling network resulting in cell cycle arrest and apoptosis [1]. Since chemotherapy has been used in tumor treatment, it has shown to be able to substantially delay local tumor growth and reduce tumor burden, thus significantly improving the five-year survival period of cancer patients. Unfortunately, tumor cells often develop multidrug resistance (MDR) after initially responding to chemotherapy treatment, which inevitably leads to tumor recurrence [2, 3]. Esophageal squamous cell carcinoma (ESCC) comprises 90% of esophageal cancer in China [4]. The 5-year survival rate is approximately 15%-25% for patients undergoing the current standard of care in the cancer clinic [5]. MDR has been widely recognized as an essential contributor to the high recurrence rate of ESCC and metastasis, causing a 90% incidence in treatment failures [6].

The mechanisms of MDR include elevated metabolism of xenobiotics, enhanced efflux of drugs, increased secretion of growth factors, inhibited apoptosis pathways, and multiple genetic factors [7]. Different cell types within the same tumor can have any MDR mechanisms, or a single tumor cell can simultaneously present a variety of resistance mechanisms making treatment regimens even more difficult [8]. Since MDR was discovered, different solutions have been proposed clinically to reduce the impact of MDR in cancer treatment. One of the solutions adopted is combination therapy using multiple chemotherapeutic agents targeting non-overlapping mechanisms. However, combination therapy has led to more complex treatment options, high medical costs, and severe adverse effects [9]. Another option is to administer treatment with an alternative regimen, including shorter time intervals between doses or higher doses in every single treatment. However, these options can also lead to more severe adverse effects and often make it hard to manage treatment in the clinic [10, 11]. Therefore, designing novel therapies targeting the causes of MDR has become of high interest but also presents a unique challenge in current oncology research.

One strategy researchers developed to combat drug resistance was RNA interference. The first RNA interference (RNAi)-based therapy was approved in the US and Europe in 2018 [12]. RNAi therapy was designed to silence multidrug-resistant genes and specifically eliminate the development of MDR in tumor cells, thus enhancing the anti-tumor effects of chemotherapeutic drugs [13, 14]. Genetic factors of MDR are complex and driven by multiple genes, and overcoming MDR by targeting only one of these factors is unlikely [15, 16]. A second obstacle is the ability to target the delivery of RNAi to tumor cells to improve efficacy by inhibiting a variety of crucial genes that trigger multiple mechanisms of MDR [17, 18].

Recent research has proved the development of nanotechnology could provide a better carrier platform for cancer treatment [19]. NPs can overcome the limitation of anti-tumor drugs by increasing the solubility of drugs and reducing toxicity to healthy tissues [20]. NPs also enhance the effects of drug treatment in tumors by improving penetration and retention [21, 22]. As for siRNA, NPs can prevent the degradation of siRNA by nuclease, thus prolonging the circulation time of siRNA in vivo [23]. In addition, NPs can also prevent drugs and siRNA from being absorbed by reticuloendothelial cells and accumulating in organs such as the liver, kidney, and spleen at high concentrations, resulting in severe toxicity side effects [24]. Most of all, NPs can further enhance the accurate delivery of siRNA and chemotherapy drugs through targeted modification, improving the outcome of anti-tumor therapy [25]. Although NP-mediated combination therapy is a promising cancer treatment, and studies have shown that the delivery systems co-loaded with drugs and siRNAs could reduce drug resistance of tumor cells [26–28], these combination therapies only silence one drug resistance gene, resulting in the MDR not being eliminated.

Here we used NPs to solve MDR in ESCC chemotherapy. We established an ESCC cell line resistant to the chemotherapy drug Adriamycin to identify two highly expressed genes, the major vault protein (MVP) and the B-cell lymphoma-2 (BCL2) gene related to MDR [29, 30]. We designed a novel self-assembling nanoparticle to deliver RNAi targeting the expression of these two genes, and Adriamycin; the design can serve as an alternative chemotherapeutic agent used in treating resistant ESCC for effective killing of the chemotherapy-resistant ESCC cells in vitro and in vivo.

## Materials and methods

### Cell line and cell culture

ESCC KYSE510 cells(510 cells)were purchased from Expasy (https://www.expasy.org, CVCL_1354). The Adriamycin-resistant cell line (510 K cells) was established from parent 510 cells by exposure to chemotherapy reagent Adriamycin (Selleck Chemicals, Houston, TX). Details can be found in Additional file 1: Materials and methods. The 510 and 510 K cells were cultured in RPMI 1640 medium supplemented with 10% FBS and 1% penicillin/streptomycin. The 510K and 510 cell lines were cultured at 37 °C in 5% CO2 in a humidified atmosphere.

### Drug-resistant cell detection

1 × 10^4^ 510 cells and 510K cells were inoculated into a 96-well microtiter plate and attached for 24 h. Different concentrations (0.03125, 0.0625, 0.125, 0.25, 0.5, 1, 2, 4, 8, 16 μg/ml) of Adriamycin-containing culture medium was added into the wells in triplicate. After incubation for 24 h, 20μL MTS solution (Sigma, St. Louis, MO) was added to each well, and the plate was incubated at 37 °C for another hour. Optical density was recorded at 490 nm using a microplate reader. Cell viability was calculated as the percentage of untreated control cells.

### Screening of multidrug resistance genes

2 × 10^5^ 510 cells and 510 K cells were collected and the total RNA was extracted using Trizol and reverse transcribed into cDNA by reverse transcriptase. The qPCR premix was prepared on ice for each qPCR run by combining 10 pmol of the primers (glutathione S-transferase π, GST-π; P-glycoprotein, P-gp; Multidrug resistance-associated protein, MRP; MVP and BCL2) with 3–4 mM MgCl_2_ and 1xSYBR Green I solution. 2 uL of cDNA was added to 18 uL of the qPCR premix. The thermal cycling conditions include the initial denaturation step at 95 °C for the 30 s, 40 cycles at 95 °C for 5 s, 58 °C for 5 s and 72 °C for 10 s. We used Glyceraldehyde 3-phosphate dehydrogenase (GAPDH) as an endogenous housekeeping gene as a standardized internal control, and each sample was run in triplicate. Please refer to the supplementary table 1 for specific primer information.

### Validation of designed siRNAs knockdown efficiency in vitro

2 × 10^5^ 510 K cells were inoculated into a 6-well plate and cultured for 24 h for attachment. Then cells were transfected with MVP-NCsiRNA, MVP-siRNA1, MVP-siRNA2, MVP-siRNA3, BCL2-NC-siRNA, BCL2-siRNA1, BCL2-siRNA2, BCL2-siRNA3 (siRNA 100 nM) by Lipofectamine™ 2000 (Invitrogen) according to the mass ratio of 1:1 (siRNAs were purchased from Shanghai GenePharma Co., Ltd). After 6 h of cultivation, the medium was changed and cultured for 48 h. Cells were collected and the total RNA and protein were extracted, respectively. Then the silencing effect of siRNAs was detected by qPCR and Western blot. Details were in Additional file 1: materials and methods.

### Preparation of CEAMB NPs

The general synthesis procedure of CEAMB NPs is as follows: First, the carboxymethyl chitosan modified by histidine cholesteryl ester and EGFR monoclonal antibody (CHCE) was synthesized according to the method in supporting materials. The infrared spectra of CHCE indicated that the CHCE had been successfully synthesized (Additional file 1: Fig. S1). 10 mg of CHCE was dissolved in 5 ml PBS buffer (20 mM, pH 7.4). Then, a probe-type ultrasonic processor is used for ultrasonic treatment at 100W for 2 min, and the ultrasonic treatment step is repeated three times. To prevent the sample solution from accumulating heat during ultrasonic treatment, we used the pulse function with 2 s on and 2 s off. Mix a specific concentration of CHCE solution, siRNA, and Adriamycin solution according to a certain mass ratio (29:2:1) at 4 °C for 30 min to synthesize the CEAMB NPs with active targeting and pH-responsive protonation through self-assembly.

### Characteristics of CEAMB NPs

The morphology of CEAMB NPs: 2µl of dissolved liquid of CEAMB NPs were dripped in the silicon wafer was dried naturally, sprayed with gold for 10s, then the morphology of CEAMB NPs was observed under a scanning electron microscope. Particle size distribution and Zeta potential distribution of CEAMB NPs in PBS solutions with different pH values: the CEAMB NPs were diluted in PBS solution with pH 7.4, 6.5, or 5.4, the particle size distribution, and Zeta potential distribution were determined by dynamic light scattering (DLS) using a Zetasizer Nano ZS90 (Malvern Instruments, UK).

Serum stability of CEAMB NPs**:** CEAMB NPs were incubated in 37 °C, 50% fetal bovine serum. At different time points (0, 6, 12 h), the blood serum stability of CEAMB NPs was determined by dynamic light scattering (DLS) using a Zetasizer Nano ZS90 (Malvern Instruments, U.K) and scanning electron microscope.

### Verification of NP tumor-specific targeting

1 × 10^5^ 510 K cells were inoculated on 12-well plates, and cultured for 24 h to adhere to the wall. Four groups of cells were incubated with siRNA, Adriamycin, CAMB NPs, CEAMB NPs (siRNA modified by Cy3, siRNA concentration is 100 nM, Adriamycin concentration is 0.5 μg/mL) respectively and incubated at room temperature for 1 h. The cells were then collected and adhesion efficiency was analyzed by flow cytometry (BD Accuri™ C6 Plus). Untreated cells were used as blank control.

### Cellular uptake of CEAMB NPs

1 × 10^5^ 510K cells were inoculated on a 12-well plate and cultured for 24 h for attachment. Cells were then incubated with different concentrations of CEAMB NPs (0, 1, 2, 4, 8, 16 μg/ml) for 4 h. Cells were collected and the cellular uptake efficiency of CEAMB NPs was detected by flow cytometry (Cy3 modified siRNA). For the laser confocal microscope detection, 1 × 10^5^ 510K cells were inoculated on a 12-well plate with a glass bottom and incubated with different concentrations of CEAMB NPs (1, 2, 4, 8, 16 μg/ml) for 4 h. The cells were then fixed with 4% paraformaldehyde for 5 min, then stained with DAPI (Invitrogen, Waltham, MA) for 10 min, and finally, images were taken using a confocal laser microscope (ZEISS).

### Intracellular delivery and lysosome escape of CEAMB NPs

1 × 10^5^ 510 K cells were inoculated on 12-well plates with a glass bottom and incubated with 16 μg/mL CEAMB NPs at five different time points (30 min, 1, 2, 3, and 4 h). The cells were fixed with 4% paraformaldehyde for 5 min, washed twice with PBS, and stained with DAPI for 10 min. The images were taken using laser confocal microscopy, and the accompanying software (ZEN3.0) was used for data analysis. For the lysosomal escape studies, the cells were stained with lysosomal marker Lysotracker (Beyotime, Shanghai, China) for 10 min. The images were taken using laser confocal microscopy, and the accompanying software (ZEN3.0) was used for data analysis.

### Detection of RNAi knockdown efficiency with NPs

2 × 10^5^ 510K cells were cultured in 6 well plates. The PBS, CEA NPs, CEAM NPs, CEAB NPs, and CEAMB NPs were incubated with cells for 48 h (siRNA concentration is 100 nM, Adriamycin concentration is 0.5 μg/mL). Cells were collected and the total RNA and proteins were extracted, respectively. RT-qPCR and western blot were used to detect the efficiency of gene knockdown. (Experimental operations were in supporting methods).

### Cell viability post in vitro nanoparticle treatment

1 × 10^4^ 510 K cells were incubated with PBS, CEA NPs, CEAM NPs, CEAB NPs, and CEAMB NPs for 48 h in a 96-well microtiter plate, three replicate holes for each NPs (siRNA concentration is 100 nM, Adriamycin concentration is 0.5 μg/mL). The culture medium was removed, and 20 μL of MTS solution was added to each well. The plate was incubated at 37 °C for another hour. Optical density was recorded at 490 nm using a microplate reader. Cell viability was calculated as the percentage of untreated control cells.

### Cell cycle and apoptosis

2 × 10^5^ 510 K cells were incubated with PBS, CEA NPs, CEAM NPs, CEAB NPs, and CEAMB NPs for 48 h (siRNA concentration is 100 nM, Adriamycin concentration is 0.5 μg/mL). For cell cycle studies, the cells were collected and fixed, using 200 μL of pre-cooled PBS and 800 μL of pre-cooled absolute ethanol and then incubated at 4 °C for 30 min. Cells were washed with PBS three times and 500ul of PBS was used to resuspend cells. Then 1ul RnaseA (Sigma, St. Louis, MO) was added and incubated for 30 min at 37 °C, 1 μL PI was then added and incubated at room temperature for 30 min for nuclear staining. Then flow cytometry was used to determine the cell cycle. For cell apoptosis studies, the cells were collected and resuspended in 100 μL of binding buffer. 10 μL 20 μg/mL FITC-modified Annexin-V (vazyme, Nanjing, China) was added and incubated at room temperature for 30 min in the dark. 5 μL of 50 μg/mL PI solution was then added (Invitrogen, Waltham, MA) and incubated at room temperature in the dark for 5 min. Then, 400 μL of binding buffer was added and detected by flow cytometry within 1 h. Single-stained annexin V-FITC and PI cells were used as positive controls.

### Biodistribution and anti-tumor of CEAMB NPs In vivo

510 K cells were harvested with trypsin when cells were 90% confluent; and diluted with serum-free 1640 medium at the concentration of 5 × 10^7^ cells /ml. 1 × 10^7^ cells (200 μL) were injected subcutaneously into 5–6-week-old nude mice. For biodistribution studies, the pure siRNA/pure Adriamycin, CHC/Adriamycin/MVP-siRNA/BCL2-siRNA NPs (CAMB NPs), and CEAMB NPs (siRNA modified by Cy3) were administrated into tumor-bearing mice via tail vein injection, respectively. Images were taken on an IVIS imaging system (PerkinElmer, Akron, OH) at 6 h post-injection. The mice were then sacrificed, and tumors and other major organs (heart, liver, spleen, lung, and kidney) were collected for ex vivo imaging. For the anti-tumor of CEAMB NPs studies***,*** when tumor size reached about 100 mm^3^, the PBS, CEA NPs, CEAM NPs, CEAB NPs, and CEAMB NPs were administrated into mice via tail vein injection. The injections were repeated on the 1st day and 18th day. Then the mice were sacrificed on the 39th day, and the tumors and other major organs were collected. The volume and weight of nude mice were measured once every three days. The tumor volume was calculated by the formula V = LW^2^/2. L and W represent the length and width of the tumor, respectively. The tumors and main organs, including the heart, liver, spleen, lung, and kidney, were collected, and tissue sections were obtained by paraffin embedding, and then H&E staining was performed. The expression of MVP, BCL2, and caspase3 protein were detected by immunohistochemistry in the tumors tissue (Experimental details in supporting methods).

### Statistical analysis

All the experiments were repeated more than three times, and the obtained data were expressed as mean ± s.e.m. An independent sample t-test statistically analyzed the data, and the results showed a significant difference (p < 0.05). The analysis was done by using GraphPad Prism software (Version 5).

## Results and discussion

### Screening of multidrug resistance genes and selecting siRNA target sequences in ESCC cells

**Fig. 1:**
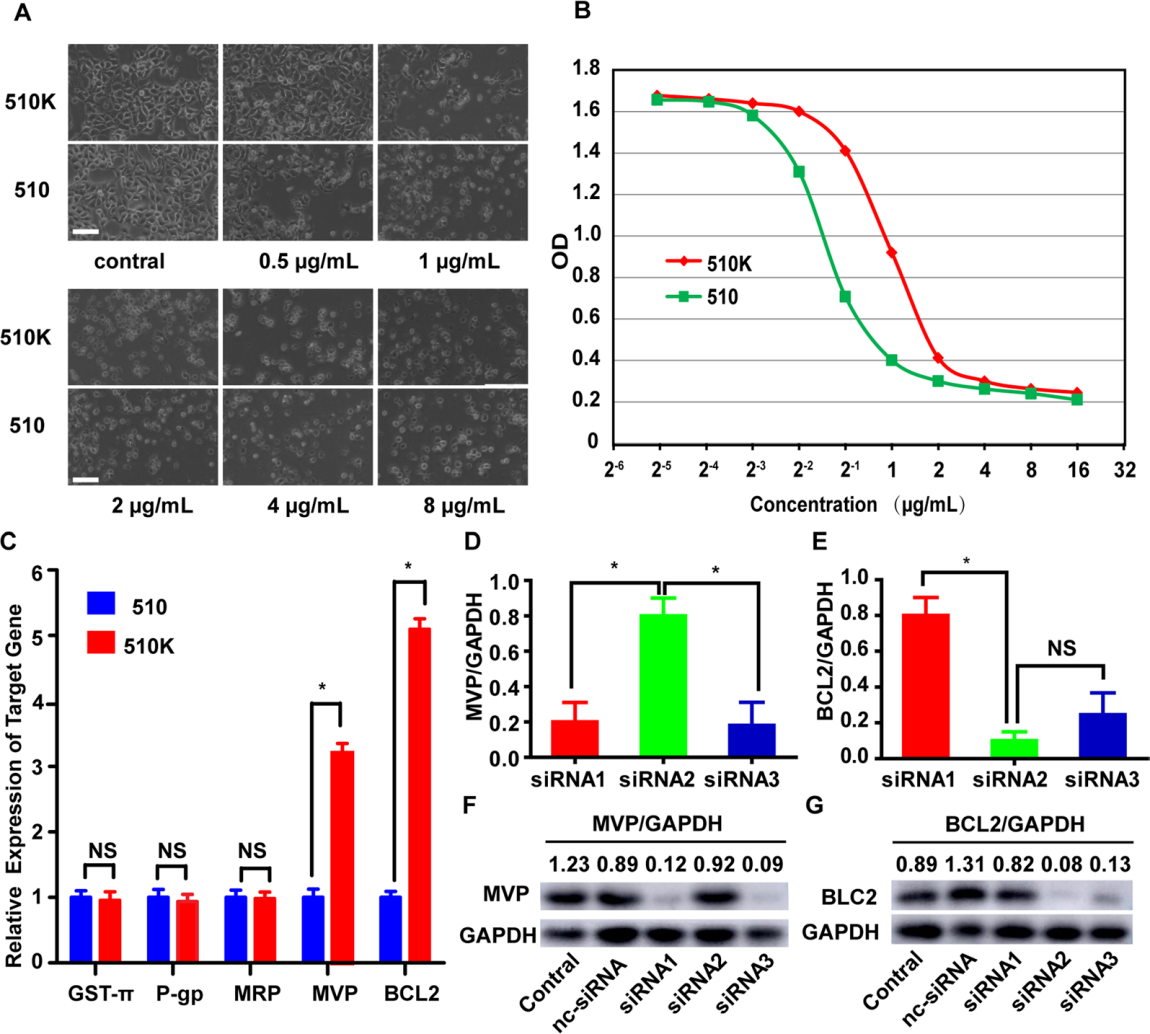
The detection of multidrug resistance genes and the multidrug resistance gene siRNA sequence. **A** Images illustrating cytotoxicity in 510K versus 510 cells using increasing concentrations of Adriamycin (0.5–8 µg/ml). Scale bar, 100 µm. **B** Spectrophotometric analysis showing cytotoxicity of Adriamycin at the variable concentration on 510K and 510 cells (mean ± s.e.m., n = 3). **C** Transcription levels of the five MDR genes in 510K and 510 cells (mean ± sem,n = 3). **D**, **E** The efficiency of silencing MVP and BCL2-mRNA by designed sequences of MVP-siRNAs **D** and BCL2-siRNAs **E** (mean ± sem, n = 3). **F**, **G** MVP and BCL2 protein expression by designed MVP-siRNAs (**F**) and BCL2-siRNAs (**G**). NS: no significant difference. *p < 0.05, double-tailed t-test

The KYSE-510 cell line is an esophageal squamous cell carcinoma and is widely used in the study of the pathogenesis of ESCC. To screen for potential MDR genes, we established an Adriamycin-resistance cell line, named 510K, from KYSE510 cells. The cell viability results indicated that the 510K cells developed a stronger drug resistance than 510 cells (Fig. 1A, B). To discover the potential MDR mechanism of ESCC, we selected five extensively studied genes related to MDR as screening targets, glutathione S-transferase π, GST-π; P-glycoprotein, P-GP; Multidrug resistance-associated protein, MRP; MVP and BCL2 [31, 32]. The results of qPCR and Western blot showed that the expression levels of MVP gene and BCL2 gene in 510 k cells were significantly increased (Fig. 1C), which indicates that these two genes may play a vital role in the MDR of ESCC. Our findings are consistent with previous studies of these two genes in MDR of other tumors [33, 34]. According to MVP-mRNA and BCL2-mRNA gene sequences, we designed and verified siRNA that can effectively silence the MVP-mRNA (Fig. 1D, F) and BCL2-mRNA (Fig. 1E, G). In the subsequent experiments, we choose MVP-siRNA1 and BCL2-siRNA2 to silence MVP-mRNA and BCL2-mRNA, respectively.

### Preparation of CEAMB NPs

**Fig. 2:**
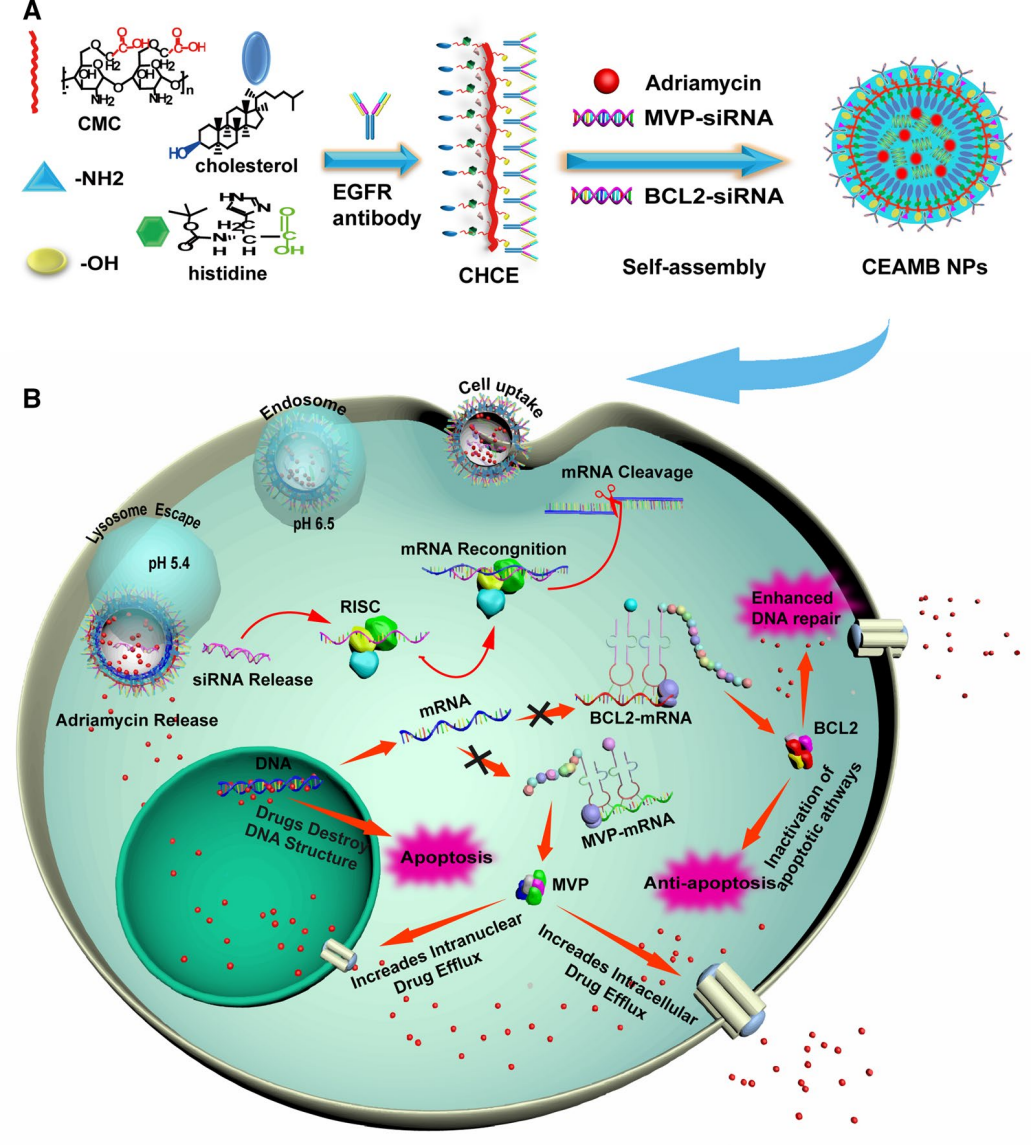
Schematic diagram of nanoparticle synthesis and intracellular delivery of CEAMB NPs.** A** Synthesis of CEAMB NPs. Carboxymethyl chitosan (CMC) containing histidine, cholesterol, and EGFR monoclonal antibodies were synthesized into CHCE, and then self-assembled with Adriamycin, MVP-siRNA, and BCL2-siRNA to form CEAMB NPs.** B** The cell uptake, intracellular transport, and anti-tumor mechanism of CEAMB NPs. The CEAMB NPs have a specific tumor-targeted delivery capability and sensitive pH-responsive protonation capability, enhancing the cellular uptake and the lysosomal escape. The CEAMB NPs can silence the MVP-mRNA and BCL2-mRNA to inhibit drug efflux and anti-apoptosis of tumor cells, enhancing the anti-tumor effect of Adriamycin

After identifying the two potentially critical genes for MDR in ESCC and selecting the RNAi sequence, we tried to eliminate ESCC resistance by suppressing their expression. To achieve a better anti-tumor effect, we prepared a novel type of CEAMB NPs with tumor targeting and pH-responsive protonation by self-assembly using CHCE, Adriamycin, MVP-siRNA, and BCL2-siRNA (Fig. 2A). The CEAMB NPs co-deliver MVP-siRNA, and BCL2-siRNA to silence MVP-mRNA and BCL2-mRNA simultaneously, inhibiting drug efflux and anti-apoptosis of tumor cells and enhancing the anti-tumor effect of Adriamycin (Fig. 2B).

**Fig. 3:**
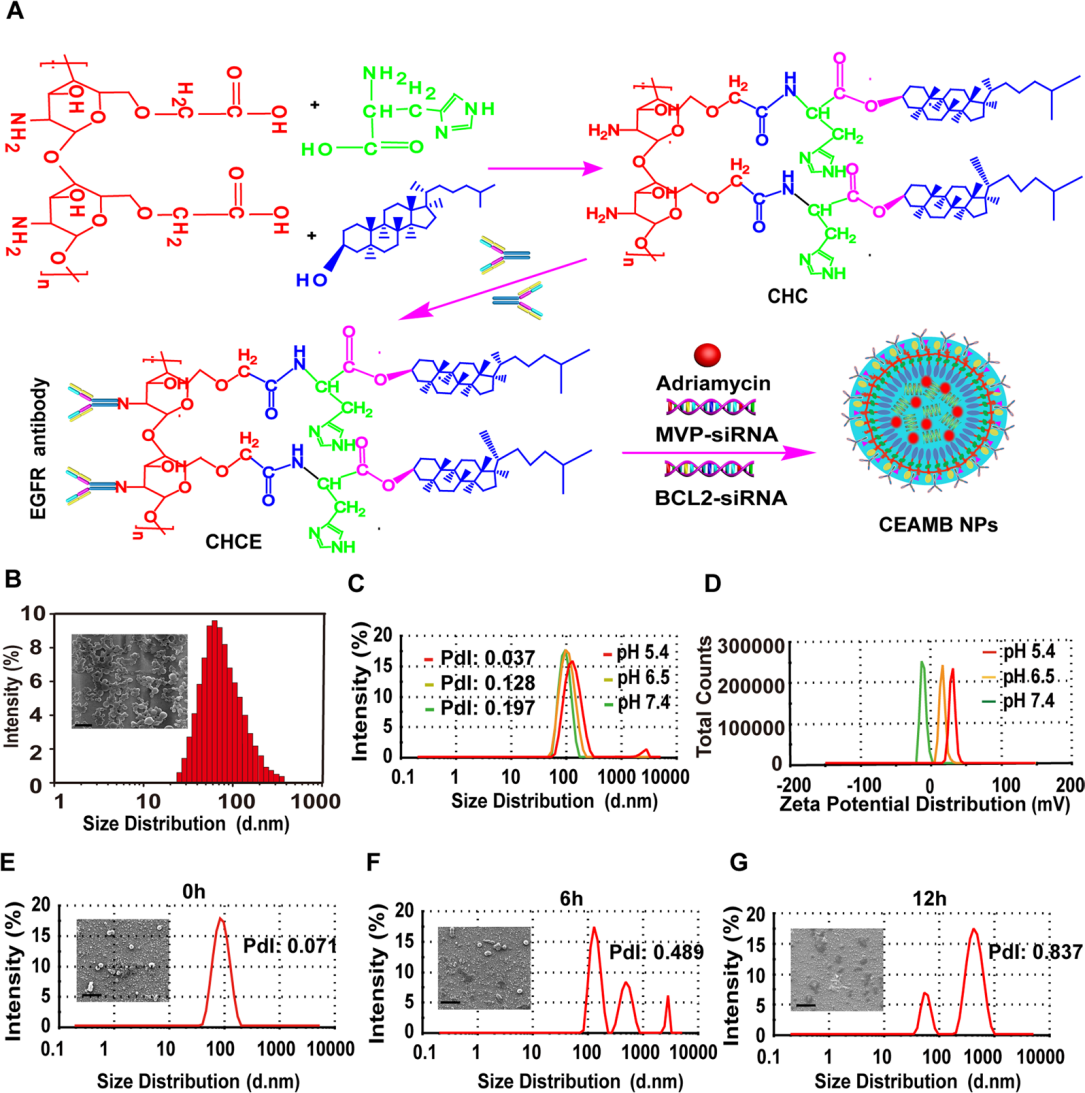
Preparation and characterization of CEAMB NPs. **A** Schematic diagram of the chemical method for the synthesis of CEAMB NPs. The CHCE was generated and then self-assembled with siRNA and Adriamycin to generate CEAMB NPs. **B** The images and particle size distribution of CEAMB NPs. Scale bar, 400 nm. Zeta potential distribution **C** and size distribution **D** of CEAMB NPs in PBS buffers with different pH. **E**–**G** The particle size distribution and morphology of CEAMB NPs in 50% serum at 0, 6, and 12 h. Scale bar, 200 nm. (The full pictures by SEM are shown in Additional file 1: Fig. S4)

In synthesizing CEAMB NPs, we first synthesized the carboxymethyl chitosan polymer modified with EGFR monoclonal antibody and histidine cholesteryl ester (CHCE) (Additional file 1: Fig. S1) and next synthesized the CEAMB NPs from CHCE, Adriamycin, MVP-siRNA, and BCL2-siRNA through self-assembly (Fig. 3A). As an amine-containing hydrophilic polysaccharide, carboxymethyl chitosan is an ideal material with excellent biocompatibility, biodegradability, and low immunogenicity [35]. As a component of the cell membrane, the cholesterol itself is highly biocompatible and may enhance the cellular uptake of the NPs [36]. The imidazole group in the histidine located at the inner surface of the NPs renders the NPs with an ability to respond to pH changes and produce a proton sponge effect to facilitate cargo siRNA escape from the endosome [37]. Studies by other researchers have found that EGFR is expressed at a higher level in ESCC compared with normal tissues, and its expression on the cell surface is a potential target molecule [38, 39]. Therefore, we coupled the designed nanoparticles with EGFR antibodies to achieve specific esophageal squamous cell carcinoma delivery.

Modification with the histidine-cholesteryl ester can form a hydrophobic domain, promoting the long-chain carboxymethyl chitosan polymer in the stretched state to curl and form encapsulated NPs [40, 41]. In addition, the lower critical micelle concentration (0.1 mg/mL) also indicated that the CHCE had a better ability to self-assemble into spheres and solid stability (Additional file 1: Fig. S2). In this experiment, we also tested the encapsulation efficiency of siRNA and Adriamycin. The results showed that the encapsulation efficiency of Adriamycin reached 95%, and the encapsulation efficiency of siRNA reached 85% (Additional file 1: Fig. S3A, B).

### Characteristic of CEAMB NPs

The CEAMB NPs had a spherical structure and lower size distributions (Fig. 3B). The size distributions of CEAMB NPs in different pH environments (90.26, 100.2, and 129.1 nm) indicated that the particle size of CEAMB NPs had little change in different pH environments (Fig. 3C). The PDI also fully indicated that CEAMB NPs had better stability in different pH (Fig. 3C). Furthermore, the Zeta charges reversal of CEAMB NPs at different pH (-8.6 mv, 17 mv, and 30 mv) indicated that CEAMB NPs have sensitive pH-responsive protonation ability (Fig. 3D). Next, we measured the serum stability of CEAMB NPs in serum, the average size distribution at 0, 6, and 12 h were about 82, 218, and 450 nm, respectively, which indicated that the CEAMB NPs could swell and become larger in volume over time. However, the morphology images showed that almost all CEAMB NPs still maintained the spherical contour, indicating that CEAMB NPs had good stability and could protect siRNA from nuclease degradation within 12 h (Fig. 3E–G). We also tested the stability of CEAMB NPs in serum by gel blocking experiment. The results showed that as the time of CEAMB NPs in the serum increased, more siRNA was released, and the siRNA was almost wholly released at the 12th hour (Additional file 1: Fig. S5). According to the above results, the total dissociation time of CEAMB NPs in serum was about 12 h, which indicated that the CEAMB NPs had good stability and could protect siRNA from nuclease degradation within 12 h. Compared with siRNA and chemotherapeutics alone, the CEAMB NPs can effectively prolong the circulation time of siRNA and chemotherapeutics in vivo. The above experimental results proved that the CEAMB NPs had better physical and chemical properties: better stability, appropriate particle size, and sensitive pH-responsive protonation ability.

These excellent physical and chemical characteristics could endow CEAMB NPs with higher delivery efficiency. The better stability can protect the integrity of the biological structure of siRNA and prevent the dissociation and release of siRNA, causing severe side effects during the delivery process [42]. Appropriate particle size can reduce the clearance efficiency of the kidney to the NPs and improve the ability of NPs to pass through the vascular endothelial space and dense extracellular matrix [43]. Sensitive pH-responsive protonation can make the surface charge of the NPs with the change of environmental pH. In the blood circulation system (Ph7.4), the surface of the NPs has a low negative charge, which can prevent the protein opsonin from adhering to the NPs and prevent the NPs from being quickly cleared by the mononuclear macrophage system [44]. In the acidic environment of tumor tissue (pH 6.5), the surface charge of the NPs is transformed into a positive charge, which promotes the cellular adhesion, uptake, and lysosomal escape of the NPs [45].

### Cellular adhesion and uptake of CEAMB NPs

**Fig. 4:**
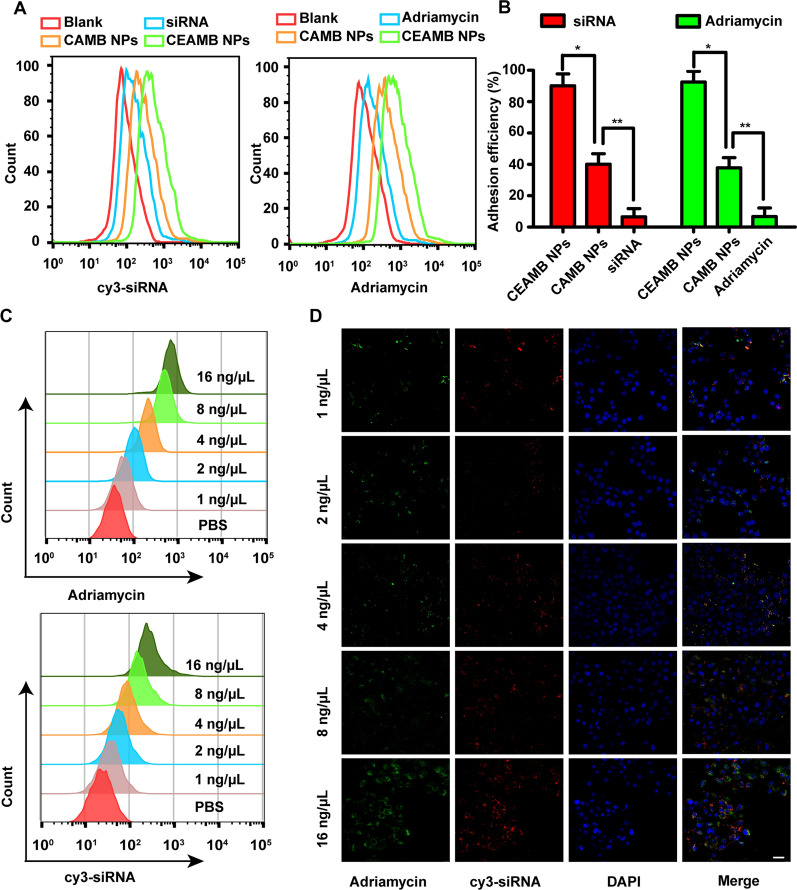
The detection of targeted adhesion and cellular uptake of CEAMB NPs in 510K cells. **A** Cells were incubated with different reagents, including blank, siRNA, CAMB NPs, CEAMB NPs, and cell adhesion was detected using Flow cytometry. Cy3-siRNA (excitation wavelength 532 nm), Adriamycin (excitation wavelength 488 nm). **B** The tumor cell adhesion efficiency of different reagents. *p < 0.05, **p < 0.01, double-tailed t-test. **C** Flow cytometry detected the uptake of CEAMB NPs. siRNA was modified by Cy3. **D** A laser confocal microscope detected the uptake of CEAMB NPs. DAPI is blue (excitation wavelength 364 nm), and Adriamycin is green (excitation wavelength 488 nm), and SiRNA is red (excitation wavelength 532 nm). Scale bar, 20 µm

Studies have shown that the siRNA must first form an RNA complex in the cytoplasm before silencing specific target genes [46]. Therefore, the NPs must have higher cellular adhesion and uptake efficiency to successfully deliver siRNA into the cytoplasm [47]. In our design, the CHC/Adriamycin/MVP-siRNA/BCL2-siRNA NPs (CAMB NPs) were modified with EGFR monoclonal antibody, which not only endowed NPs with the ability to specifically recognize tumor cells but also triggered receptor-mediated endocytosis to promote cellular uptake efficiency of NPs [48]. The adhesion efficiency showed that compared to pure siRNA (6.5 ± 5.3%), pure Adriamycin (6.7 ± 5.5%), and CAMB NPs (siRNA: 40.1 ± 6.7%, Adriamycin: 37.8 ± 6.5%), the CEAMB NPs (siRNA: 90.1 ± 7.6%, Adriamycin: 92.5 ± 6.9%) had the best cellular adhesion ability (Fig. 4A, B).

**Fig. 5:**
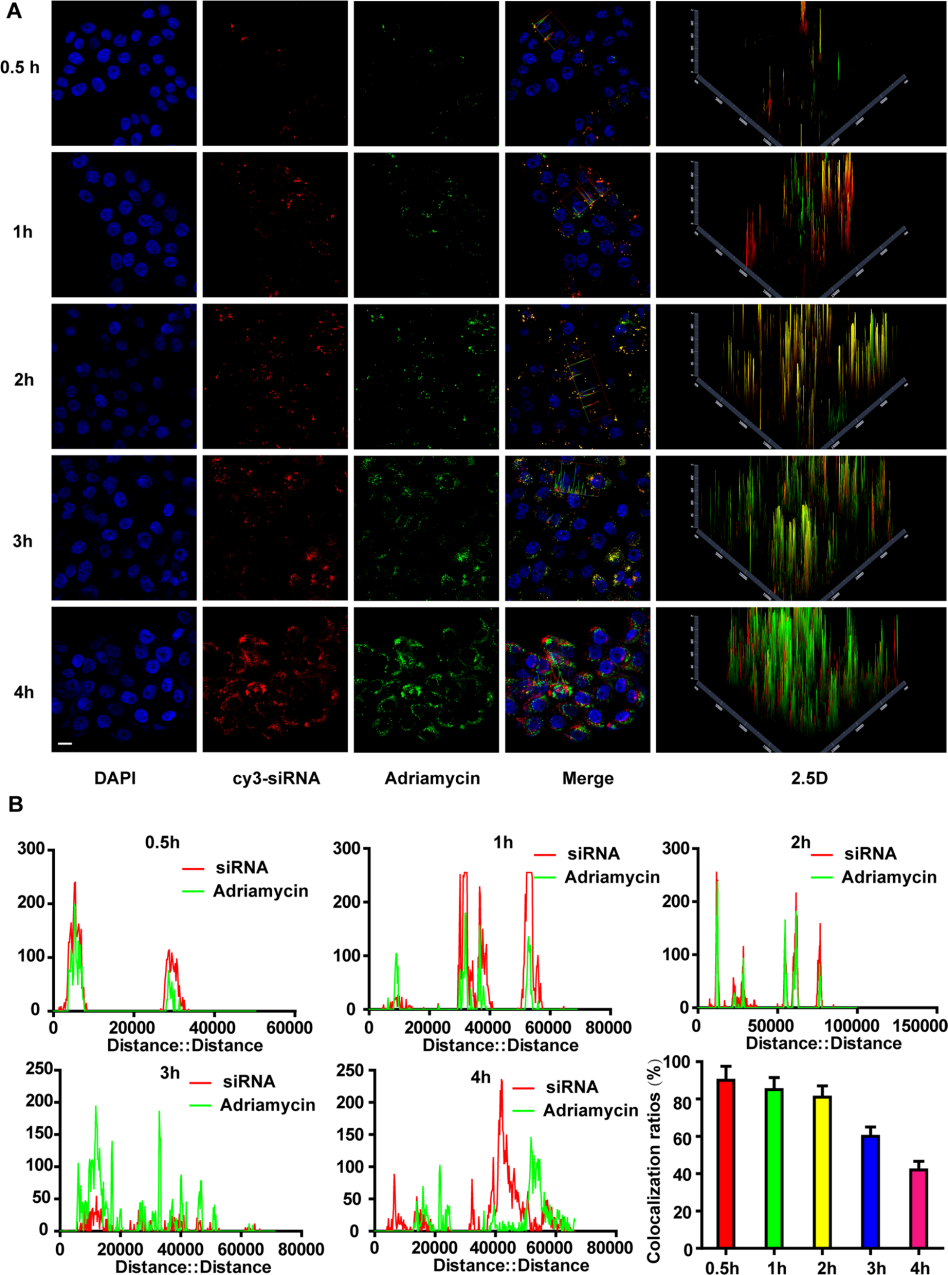
The intracellular transport of CEAMB NPs. **A** The laser confocal images and 2.5D images show the colocalization of Adriamycin and siRNA displayed by a confocal microscope at different time points post-treatment. The cells were labeled with three colors: blue (DAPI), marking the nuclei; Green, marking the Adriamycin; Red, marking the siRNA. Scale bar, 10 µm. **B** The colocalization fluorescence intensity distribution at indicated time points post-treatment and colocalization rate of Adriamycin and siRNA

Next, we detected the cellular uptake of CEAMB NPs; the results showed that with the increase of the concentrations, the uptake efficiency of siRNA and Adriamycin also increased, and the increased efficiency of both siRNA and Adriamycin were consistent (Fig. 4C). The fluorescence images also showed that siRNA and Adriamycin were spatially consistent in tumor cells, which indicated that the NPs had good stability in cellular uptake and could ultimately deliver siRNA and Adriamycin into cells through cell membranes (Fig. 4D). Then we observed the fate of CEAMB NPs co-delivering siRNA and Adriamycin in cells. The fluorescence images showed that with the increase of time, the more siRNA and Adriamycin uptake by tumor cells (Fig. 5A). The colocalization rate of Adriamycin and siRNA in cells showed that Adriamycin and siRNA were tightly combined in the early stage (1 h: 90 ± 5%, 2 h: 85 ± 4%), and then gradually separated over time (3 h: 60 ± 5%, 4 h: 42 ± 4%) (Fig. 5B). The above results indicate that the CEAMB NPs had good stability and could effectively deliver siRNA and Adriamycin into the cell and release siRNA and Adriamycin into the cytoplasm.

### Lysosomal escape of CEAMB NPs

**Fig. 6:**
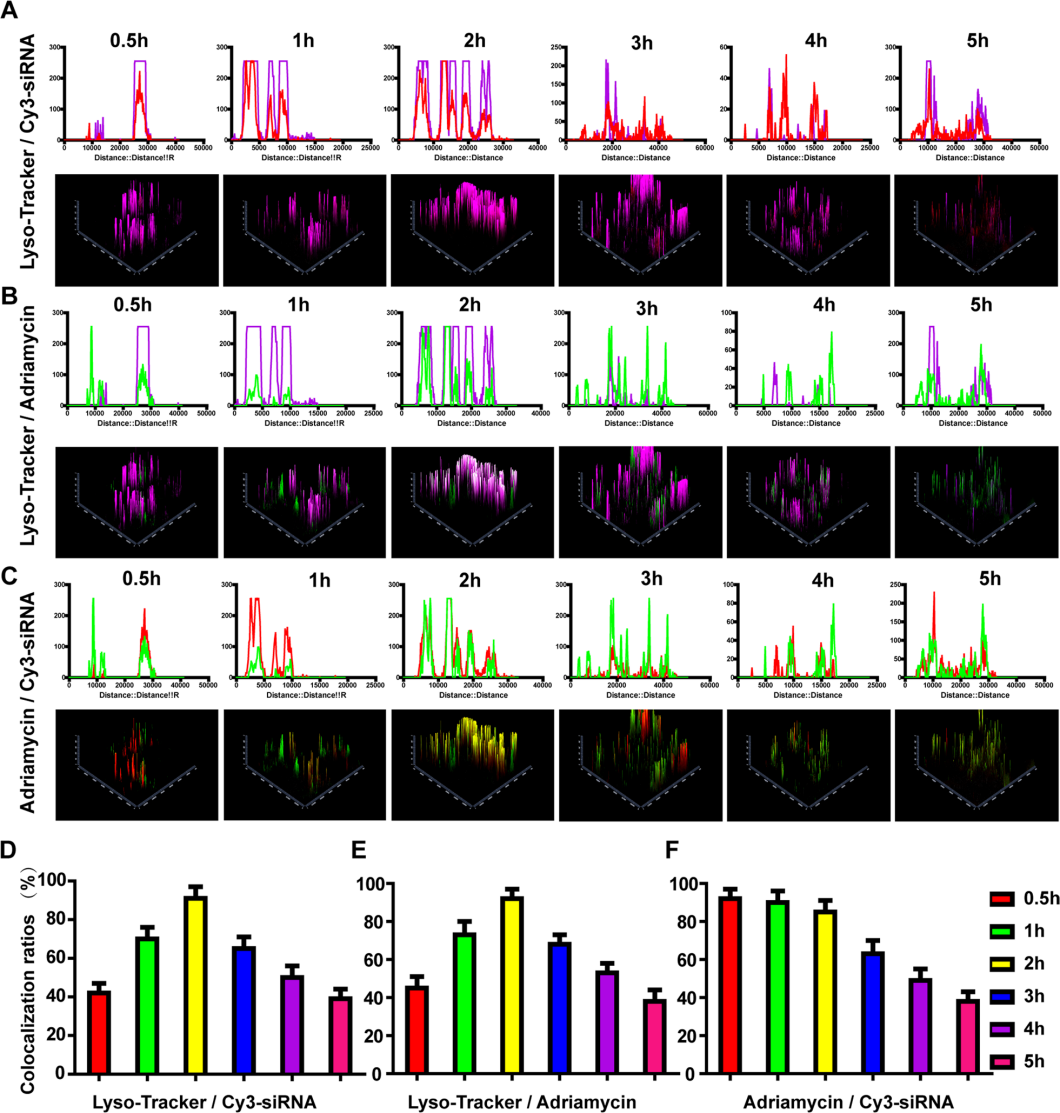
Detection of the lysosomal escape of CEAMB NPs in 510K cells (The laser confocal images are shown in Additional file 1: Fig. S6). The CEAMB NPs were incubated with 510 K cells. Next, the colocalization fluorescence intensity distribution and 2.5D images were taken for the colocalization of lysosome and siRNA (**A**), lysosome and Adriamycin (**B**), Adriamycin and siRNA (**C**), respectively. The observed targets were labeled with three colors: Adriamycin is green, siRNA is red, and lyso-tracker is purple. The degree of the colocalization was measured at the indicated time points with groups of lysosome and siRNA (**D**), lysosome and Adriamycin (**E**), Adriamycin, and siRNA (**F**)

As an important biological barrier in the process of NPs delivery, lysosomes play an important role in limiting the delivery efficiency of NPs [49]. Therefore, the lysosomal escape ability of NPs determines its ultimate delivery efficiency. To enhance the lysosomal escape ability, we designed and endowed the CEAMB NPs with histidine cholesteryl ester, which could induce the protonation sponge effect of lysosomes, thus causing instability of lysosomal membrane or rupture of lysosomes [50]. The colocalization rate of Adriamycin and lysosomes (0.5 h: 45.6%, 1 h: 73.7%, 2 h: 92.5%, 3 h: 68.5%, 4 h: 53.3% and 5 h: 45.6%) (Fig. 6A, D), siRNA and lysosomes (0.5 h: 42. 5%%, 1 h: 7.6%, 2 h: 91.6%, 3 h: 65.6%, 4 h: 50.6% and 5 h: 39.5%) (Fig. 6B, E) showed that the CEAMB NPs entered in lysosomes at early and reached the maximum at about 2 h, then gradually separated from lysosomes and the more CEAMB NPs separated from lysosomes over time. The results could powerfully prove that the CEAMB NPs had better lysosomal escape ability. In addition, we also tested the colocalization rate of Adriamycin and siRNA. The results were consistent with the intracellular delivery results of CEAMB NPs (Fig. 6C, F). Summarizing the above results, the CEAMB NPs had better cellular uptake and lysosomal escape ability and could release siRNA and Adriamycin into the cytoplasm.

### CEAMB NPs used to effectively inhibit the expression of siRNA target genes

**Fig. 7:**
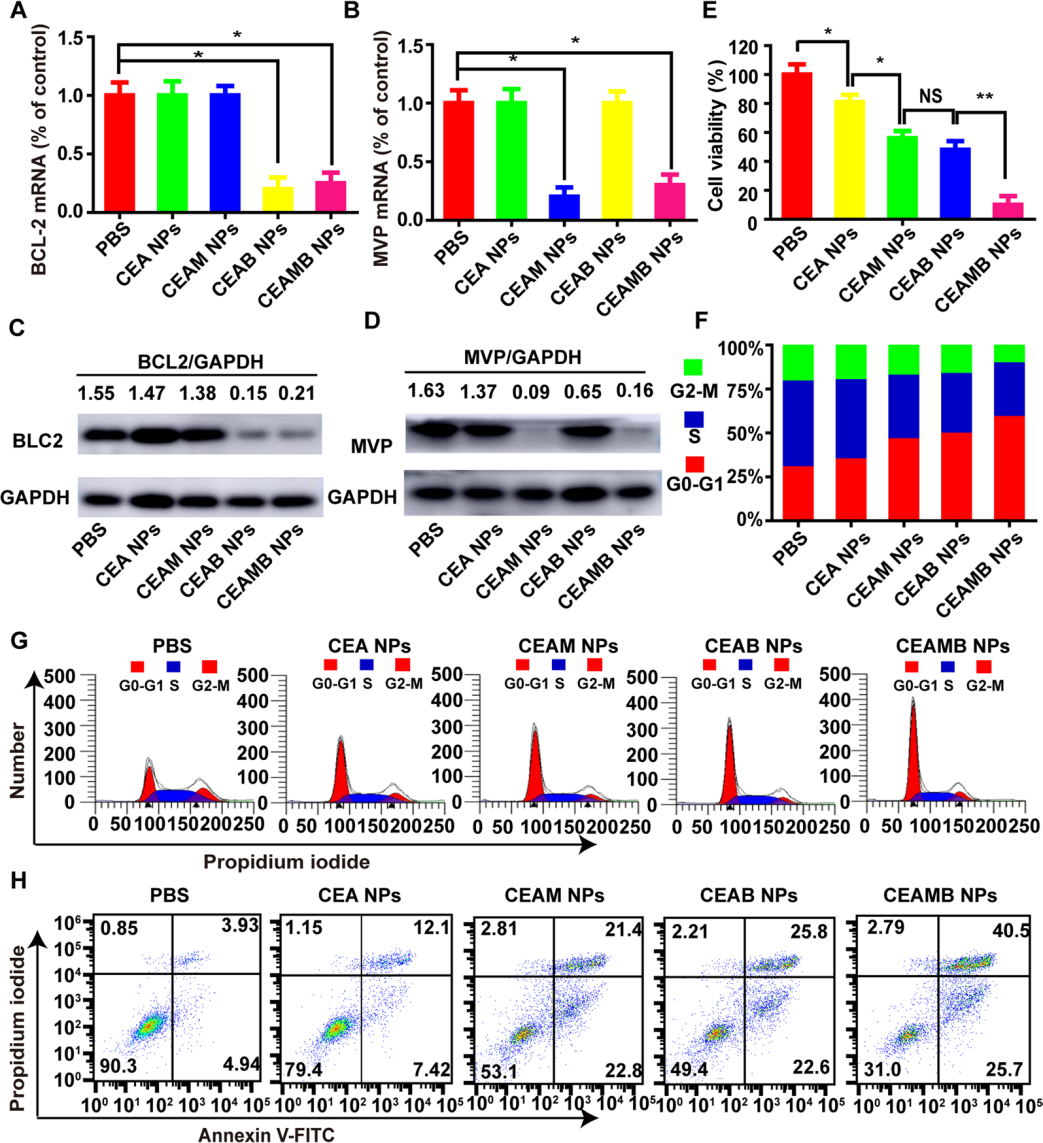
CEAMB NPs can effectively silence drug-resistance genes in 510K cells and then cause changes in cell viability, cycle, and apoptosis. 510 K cells were incubated with the control PBS and different NPs: CHCE/Adriamycin nanoparticles (CEA NPs), CHCE/Adriamycin/MVP-siRNA nanoparticles (CEAM NPs), CHCE/Adriamycin/BCL2- siRNA nanoparticles (CEAB NPs), and CHCE/Adriamycin/MVP-siRNA /BCL2-siRNA nanoparticles (CEAMB NPs). Next, the mRNA levels of MVP and bcl2 were detected by RT-qPCR respectively (**A**, **B**). (mean ± sem, n = 3). *p < 0.05, double-tailed t-test. Also, the protein of treated cells was extracted, and the expression levels of MPV and BCL2 was detected by western blot (**C**, **D**). In the above, the mRNA and protein levels of housekeeping gene GAPDH were used as normalization internal control (**E**). MTS assay was used to detect the viability ratio of 510 K cells compared with the PBS treated group (mean ± sem, n = 3). NS: no significant difference. *p < 0.05, **p < 0.01, double-tailed t-test. In all groups above, the cell cycle (**F**, **G**) and apoptosis (**H**) of 510K cells were detected by flow cytometry. The lower left quadrant, lower right quadrant, upper right quadrant, and upper left quadrant represented the survival, early apoptosis, late apoptosis, and necrosis, respectively (%)

Because the MVP gene and BCL2 gene respectively increase the drug efflux and anti-apoptosis of tumor cells, our experimental results also show that these two genes are highly expressed in Adriamycin-resistant cells. Therefore, we adopted a double sensitization strategy of co-loading NPs with MVP-siRNA and BCL2-siRNA to simultaneously silence the MVP gene and the BCL2 gene and eliminate the drug resistance of 510 K cells. The qPCR results showed that the CAM NPs and CAB NPs could only silence MVP-mRNA and BCL2-mRNA, respectively, while the CEAMB NPs could silence MVP-mRNA and BCL2-mRNA simultaneously (Fig. 7A, B). Lowered protein expression indicated by results of western blot analysis further proved that CEAMB NPs could silence MVP-mRNA and BCL2-mRNA simultaneously (Fig. 7C, D). In addition, the above results indicated that the co-loading of Adriamycin did not affect multiple siRNAs to silence the target gene.

Next, we detected the anti-proliferative ability of CEAMB NPs. The 510K cells treated with the CEAM NPs and CEAB NPs had lower activity than those treated with the CEA NPs, while the cells treated with the CEAMB NPs had the lowest activity (Fig. 7E). The results indicated that silencing MVP-mRNA and BCL2-mRNA simultaneously could more effectively eliminate multidrug resistance and promote the anti-tumor effect of Adriamycin. The cytotoxicity results further proved that the CEAMB NPs had the most effective anti-tumor ability (Additional file 1: Fig. S7A–C). Researchers have demonstrated that Adriamycin resulted in cell cycle arrest in G0/G1 phase [51], so the cell cycles were tested to detect the anti-tumor ability of CEAMB NPs. The results showed that the CEAMB NPs had the highest proportion of cells in the G0-G1 phase (Fig. 7F, G). Also, our results showed that the CEAM NPs and the CEAB NPs could induce more tumor cells into apoptosis (44.2%, 48.4%) than the CEA NPs (30.1%), while the CEAMB NPs could induce the most tumor cells to undergo apoptosis (66.2%), these findings are in line with previous research on the function of Adriamycin (Fig. 7H) [52]. The results indicated that the CEAMB NPs might eliminate drug efflux and anti-apoptotic ability of MDR 510K cells to improve the anti-tumor effect of Adriamycin, leading the most tumor cells to arrest in the G0/G1 phase and into apoptosis.

### The targeting delivery of CEAMB NPs in vivo

**Fig. 8:**
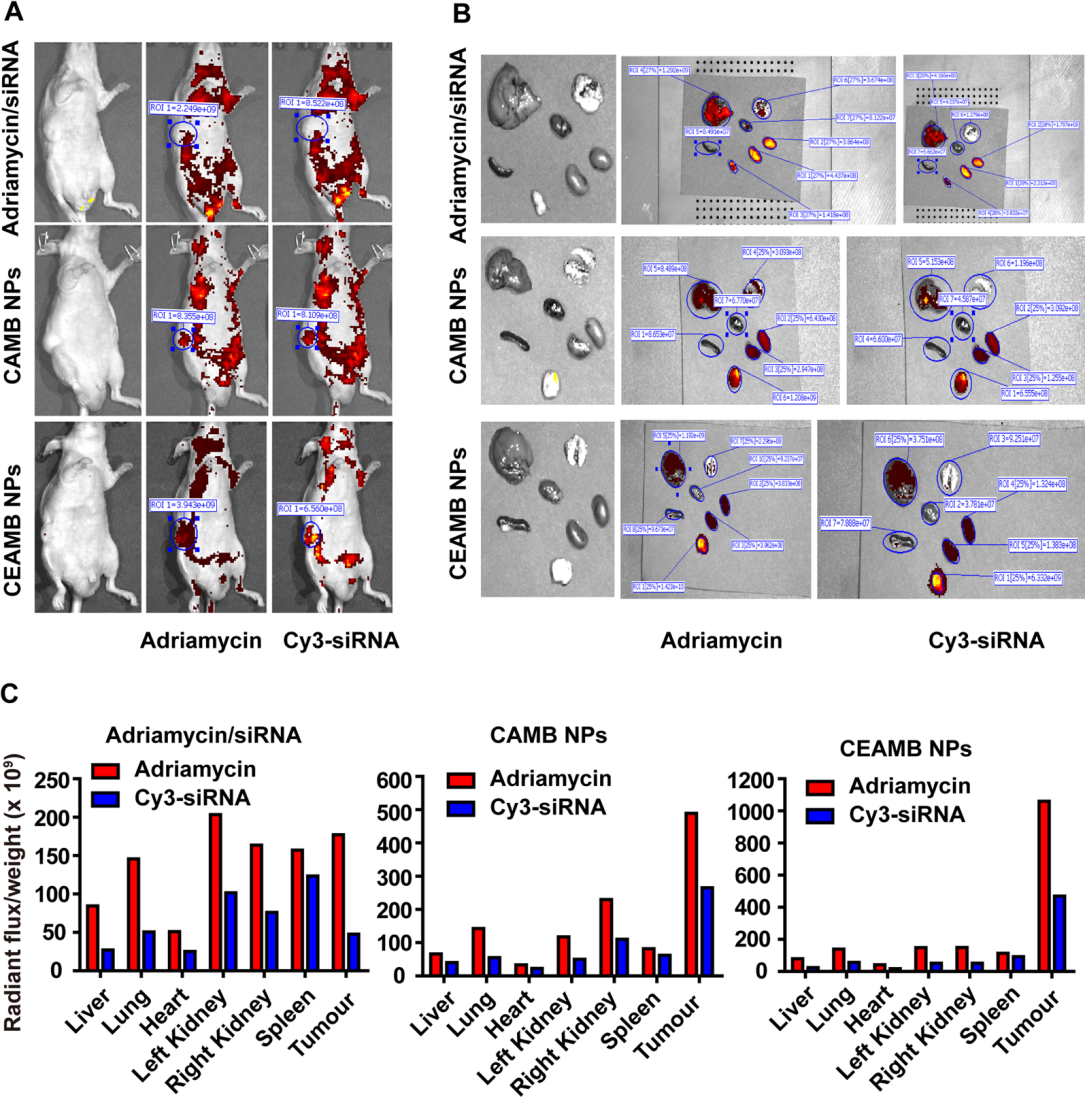
Biodistribution of CEAMB NPs in tumor-bearing mice. The 510K cells were subcutaneously inoculated in a nude mouse. CEAB NPs, CEAMB NPs, and Adriamycin/siRNA were delivered into the mouse via tail vein injection. **A** 6 h post-injection, images were taken using whole-body NIR fluorescence. **B** And then fluorescence signal of the mouse tumor and major organs (liver, lung, heart, left kidney, right kidney, and spleen) was imaged ex vivo. **C** The intensity of the fluorescence signal per emitter was quantified from the three above groups

To improve targeted delivery ability in vivo, the CAMB NPs were modified with EGFR monoclonal antibody. The fluorescence reflectance images showed that the CEAMB NPs had higher Adriamycin and siRNA accumulation concentrations in the tumor site (Fig. 8A). The organ and tumor images showed that the use of only Adriamycin and siRNA had a higher accumulation concentration in the liver and kidney and a lower concentration in tumor tissue. In contrast, both CAMB NPs and CEAMB NPs can be significantly enriched in the tumor sites of mice, and among them, CEAMB NPs have the highest degree of enrichment (Fig. 8B, C). Because EGFR is highly expressed in ESCC tumor cells, our design using EGFR antibody demonstrates that CEAMB NPs have a better tumor targeting ability as well as the ability to deliver Adriamycin and siRNA to the tumor effectively.

### The anti-tumor effect of CEAMB NPs in vivo

**Fig. 9:**
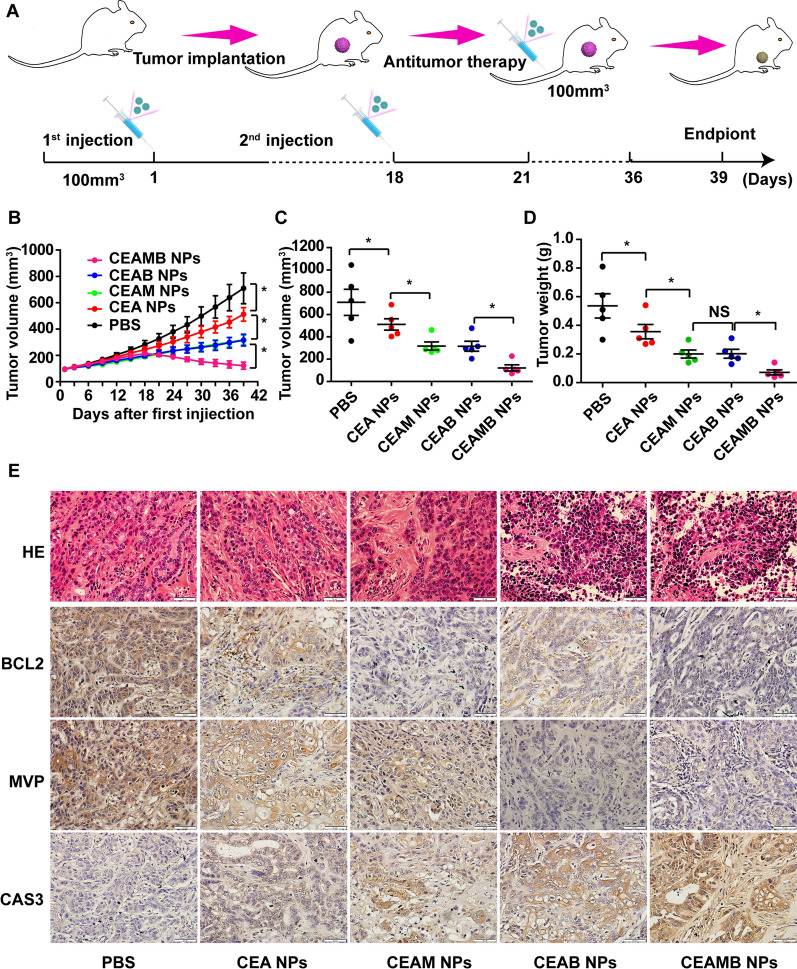
The anti-tumor effect of NPs in tumor-bearing mice. **A** Scheme of the mouse model. K510 cells were subcutaneously injected into nude mice, and tumor engraftment was monitored. When tumor size reached 100 mm^3^, the different NPs (CEA, CEAM, CEAB, CEAMB) and control PBS were injected into the tail vein on day 1 and day 18. The mice were sacrificed on day 39 for IHC. **B** The growth curves of tumors. The tumor volume was measured one time every 3 days. (mean ± s.e.m., n = 5). *p < 0.05, (two-tailed t-test). **C** The tumor volume (mm^3^) and **D** the tumor weight (g) was measured at the end of treatment. (mean ± s.e.m., n = 5). *p < 0.05, (two-tailed t-test). *NS* no significant difference. **E** The tumors of each group were removed at the endpoint. The dissected slides were used for H&E staining and detecting BCL2, MVP, and CAS3 (Caspase3) protein level by IHC. Scale bar, 100 µm

The above experiments have proven that RNAi carried by our designed NPs can effectively enrich the tumor site, therefore inhibiting the expression of the target gene. The delivered Adriamycin can trigger the apoptosis of tumor cells. Next, we tried to perform functional verification in vivo. The 510K cell-xenograft animal model was established, and the first treatment of NPs was performed when the volume of tumors was about 100 mm^3^. The second treatment was then performed on the 18th day after the first treatment (Fig. 9A). The tumor growth curve showed the CEAMB NPs, CEA NPs, CEAM NPs, and CEAB NPs could effectively inhibit tumor growth, but only CEAMB NPs could decrease tumor volume (Fig. 9B). The tumor volume and tumor mass results at the end of treatment showed the same trends as the tumor growth curve (Fig. 9C, D). These results could be directly observed from the photographs of tumor-bearing mice and tumors excised from the mice (Additional file 1: Fig. S8A). In addition, the results of the weight of the mice at the end of the treatment showed no significant difference (Additional file 1: Fig. S8B).

Meanwhile, the histopathological analysis of tissue sections isolated from the mice displayed no significant pathological changes in the heart, liver, spleen, and lung in all treated groups, revealing the safety of CEAMB NPs (Additional file 1: Fig. S8C). The above results indicated that chemotherapeutic drugs combined with multiple drug resistance gene siRNAs had a better therapeutic effect than the use of a single-drug resistance gene siRNA or chemotherapeutic drugs alone. Finally, the synergistic anti-tumor mechanisms in vivo were investigated. The results of IHC showed that the CEAMB NPs could effectively reduce the expression of MVP and BCL2 proteins via silencing their transcript mRNA, respectively (Fig. 9E). An apoptosis marker protein, Caspase3, was tested to detect the anti-tumor ability of CEAMB NPs [53]. The results showed the expression level of Caspase3 protein in the CEAMB NPs group was the highest, which indicated that CEAMB NPs could effectively induce tumor cell apoptosis in vivo.

Furthermore, apparent nuclear shrinkage, fragmentation, and absence in the hematoxylin and eosin-stained sections of tumor tissue proved that the CEAMB NPs had a better anti-tumor effect (Fig. 9E). The above results indicated that the CEAMB NPs could effectively inhibit drug efflux and anti-apoptosis of tumor cells from eliminating the MDR of tumors, thereby enhancing the anti-tumor effect of chemotherapeutics. Moreover, the dual sensitization strategy loading with multiple drug resistance gene siRNAs could effectively improve the drug's anti-tumor effect.

## Conclusions

In summary, we have prepared a new type of CEAMB NPs with targeting and pH-responsive protonation to treat the MDR of ESCC; the NPs could extend the circulation time of siRNA and Adriamycin in vivo and enhance the targeted accumulation of siRNA and Adriamycin in esophageal tumors. Furthermore, the CEAMB NPs adopted the double sensitization strategy of using RNA interference technology, which could effectively silence drug efflux gene and anti-apoptosis gene simultaneously to eliminate MDR, thus enhancing the anti-tumor effect of Adriamycin in vitro and in vivo. Therefore, we speculate that the delivery system has the potential to treat a variety of advanced cancers by the combined chemotherapy and RNA interference technology.

## Supplementary Information

**Additional file 1.** Additional materials and methods, figures and tables.

## Data Availability

The data supporting the findings of this study are available within this paper and Additional files. Additional data can also be available from the corresponding author on reasonable request.

## Abbreviations

MDR: Multidrug resistance
NP: Nnanoparticle
RNAi: RNA interference
BCL2: B-cell lymphoma-2
MVP: Major vault protein
GST-π: Glutathione S-transferase π
P-gp: P-glycoprotein
MRP: Multidrug resistance-associated protein
GAPDH: Glyceraldehyde 3-phosphate dehydrogenase
ESCC: Esophageal squamous cell carcinoma
EGFR: Epidermal growth factor receptor
DLS: Dynamic light scattering
