# Supplementary material for: Multifunctional nanoparticles co-loaded with Adriamycin and MDR-targeting siRNAs for treatment of chemotherapy-resistant esophageal cancer
Source: J Nanobiotechnology. 2022 Mar 28;20:166. doi: 10.1186/s12951-022-01377-x (PMC8962182; doi:10.1186/s12951-022-01377-x)
Supplement: Supplementary file 1 — Additional file 1. Additional materials and methods, figures and tables. [file 12951_2022_1377_MOESM1_ESM.docx]

**Additional file 1**

Multifunctional Nanoparticles Co-loaded with Adriamycin and MDR-targeting siRNAs for Treatment of Chemotherapy-resistant Esophageal Cancer

Xiangyang Zhang ^1, 2^, Min Wang ^1^, Junyi Feng ^1^, Bin Qin ^1^, Chenglin Zhang ^1^,

Chengshen Zhu ^2^, Wentao Liu ^2^, Yaohe Wang ^2^, Wei Liu ^3^, Lei Huang ^4^,

Shuangshuang Lu ^1^, Zhimin Wang ^1, *^

^1^Sino-British Research Centre for Molecular Oncology, National Centre for International Research in Cell and Gene Therapy, School of Basic Medical Sciences, Academy of Medical Sciences, Zhengzhou University, Zhengzhou, Henan Province, P . R. China

^2^School of Material Science and Engineering, Zhengzhou University, Zhengzhou, Henan Province, P. R. China

^3^Children's Hospital Affiliated to Zhengzhou University, Zhengzhou, Henan Province, P. R. China

^4^Inflammations Immunity Research Theme, Translational and Clinical Research Institute, FMS, Newcastle University, NE1 7RU, Newcastle Upon Tyne, United Kingdom

*Corresponding authors:

Zhimin Wang Email: wangzm@zzu.edu.cn

*Corresponding authors:

Zhimin Wang Email: wangzm@zzu.edu.cn

**Supplementary materials and methods**

1. **Synthesis of CHCE**

Synthesis of Boc-histidine cholesteryl ester intermediate （If not specified, all reagents are purchased from Aladdin Bio-chem Tech, Shanghai, China: Added 200 mg of cholesterol (Sigma-Aldrich, St. Louis, MO) and 130 mg of t-Butyloxy carbonyl-histidine (Sigma-Aldrich, St. Louis, MO) to 10 mL of dichloromethane (Sinopharm Chemical Reagent Co., Ltd, Shanghai, China), then added 140 mg of dicyclohexylcarbodiimide and 2 mg of 4-dimethylaminopyridine. The solution was left to react at room temperature, and after 15 hours, white precipitate was filtered off. The solvent was removed under reduced pressure and purified to obtain the Boc-histidine cholesteryl ester intermediate;

Synthesis of histidine cholesteryl ester intermediate (HIS-CHO): Dissolved 200 g Boc-histidine cholesteryl ester intermediate in 7 mL of dichloromethane, then added 1 mL of trifluoroacetic acid, and stirred for 1h, The mixture was then neutralized with 2 mL of 36.6% aqueous ammonia solution (Sinopharm Chemical Reagent Co., Ltd, Shanghai, China), and reduced. The solvent was evaporated，and the residue was diluted in ethyl acetate, and subsequently washed with water. Next the solution was dried over magnesium sulfate. The solvent was evaporated under reduced pressure again to obtain a white powdery histidine cholesteryl ester intermediate;

Synthesis of histidine cholesteryl ester modified carboxymethyl chitosan polymer compound (CHC): Dissolved 520 mg histidine cholesteryl ester in 100 mL tetrahydrofuran to prepare solution A. Next 500 mg carboxymethyl chitosan, 250 mg 1-ethyl-(3-dimethyl aminopropyl) carbodiimide hydrochloride, and 150 mg N-hydroxysuccinimide was dissolved in 50 mL 1% acetic acid. The carboxyl group was activated in solution for 1 hour at room temperature. Solution A was added by stirring, and left to react at room temperature for 72 hours. Tetrahydrofuran was then evaporated under reduced pressure, and 200 mL of absolute ethanol was added and stirred to accumulate the precipitate. The precipitate was filtered, and washed with tetrahydrofuran, acetone, and ether in turn to obtain a white powder of Histidine cholesteryl ester modified carboxymethyl chitosan polymer compound (CHC).

Synthesis of carboxymethyl chitosan polymer compound modified with histidine cholesteryl ester and targeting marker: Dissolved 38 mg of carbodiimide hydrochloride and 23 mg of N-hydroxysuccinimide in 2 mL of a 0.1 mM Anti-EGFR monoclonal antibody solution (Abcam, Cambridge, United Kingdom), The carboxyl group was activated for 1 hour to obtain activated targeting marker solution. Then 40 mg of CHC was dissolved in 10 mL of 1% hydrochloric acid solution, activated targeting marker solution was then added by stirring and left to react at 4°C for 24 hours. Freeze-drying was used to obtain carboxymethyl chitosan polymer compound modified by histidine cholesteryl ester and EGFR monoclonal antibody (CHCE).

1. **Infrared spectroscopy**

The dry product powder is mixed with dry potassium bromide (Sinopharm Chemical Reagent Co., Ltd, Shanghai, China) according to a certain proportion and fully ground, then compressed with a tablet press. Analyze the absorption peak of NPs in the wavelength scanning range of 400-4000 cm^-1^ by infrared spectrometer (Bruker Optics, Germany).

1. **Critical Micelle Concentration of CHCE**

The fluorescence probe method was used to detect the critical micelle concentration of CHCE in an aqueous solution: A 5mL acetone solution in which the concentration of pyrene is 6.0×10^-7^ mol/L was put it in an ampere flask, and left to stand to volatilize all acetone. Then 5 mL of CHCE solutions of different concentrations were added to make the final concentration of pyrene 6.0×10^-7^ Mol/L. The solution was equilibrated for 20 minutes in a water bath ultrasonic instrument. Under the conditions of excitation wavelength 335 nm, excitation slit width 5 nm, and emission slit width 10 nm, a fluorescence spectrophotometer were used to measure the fluorescence intensity of pyrene at 372 nm and 383 nm (I372 and I383) in different CHCE concentrations. The logarithm of CHCE and I372/I385 concentration were plotted, and the intersection of the two straight lines represents the critical micelle concentration of CHCE.

1. **Establishment of Adriamycin-resistant cell line**

The Adriamycin-resistance variant 510K was established from the surviving population of 510 esophageal cancer cells after treatment of the parent population with several rounds of 1μg/ml Adriamycin. According to the experimental design, each following treatment is after the cells reach an average growth rate and about 80% confluence. Once cells could be stably sub-cultured in 1μg/ml Adriamycin, the Adriamycin-resistant cell line 510K was obtained. In the follow-up work, the cells were cultured in the medium of 1μg/ml Adriamycin to maintain their drug resistance.

1. **Determine the siRNA and Adriamycin encapsulation efficiency**

Mixed CHCE solution， siRNA and Adriamycin solution according to different mass ratio (0.5:1:1, 1:1:1, 2:1:1, 4:1:1, 8:1:1, 16:1:1, 32:1:1) at 4°C for 30 min to synthesize the multifunctional nanoparticles. Then 50 µl of nanoparticles were transferred to the adsorption column and centrifuged at 12000g for 5 minutes at 4°C. The nanoparticles were retained in the adsorption column, and non-encapsulated free siRNA and Adriamycin remained in solution. Spectrophotometry was used to detect siRNA and Adriamycin concentrations at wavelengths of 260-nm and 480-nm respectively. The encapsulation efficiency of siRNA and Adriamycin were calculated as follows:

Encapsulation efficiency = (WT−WF)/WT × 100%

Where WT is the total weight of siRNA or Adriamycin, and WF is the weight of non-encapsulated free siRNA or Adriamycin.

1. **RNA Extraction, cDNA Synthesis and qPCR**

Post treatments, the cells cultured in 6 well plates were used to extract RNA. Each well was washed twice with PBS, and 200 μL of 0.25% trypsin containing 0.26 mM EDTA was added to separate cells. 800μL of medium containing serum was then added to stop the trypsin reaction. Cells were collected, centrifuged at 300 g for 5 minutes, and the supernatant was discarded. Total RNA was extracted using Trizol (Invitrogen, Carlsbad, CA) and equal amounts of cDNA were synthesized using the first-strand cDNA synthesis kit (Vazyme, Nanjing, China). All qPCR performed using SYBR Green (Applied Biosystems, Waltham, MA) was conducted at 95℃ for the 30s, 40 cycles at 95℃ for 5s, 58℃ for 5s and 72℃ for 10s. The specificity of the reaction was verified by melt curve analysis. The housekeeping gene GAPDH was amplified as an internal control. All samples were run in triplicate.

1. **Western blot analysis**

The cells were incubated in 6 well plate and all media was removed. Each well was washed twice with PBS, and 200 μL of 0.25% trypsin containing 0.26 mM EDTA was added to separate cells. 800 μL of medium containing serum was then added to stop the trypsin reaction. Cells were collected, centrifuged at 300 g for 5 minutes, and the supernatant was discarded. 200 μL of RIPA buffer (Beyotime, Shanghai, China) was used to extract protein, and then protein concentrations were calculated using a BCA Protein Assay Kit (Beyotime, Shanghai, China). An equal amount of protein was separated on SDS-PAGE and then transferred to the Hybond P membrane (Cytiva, Marlborough, MA). The membrane was blocked using a 5% skimmed milk solution at room temperature for 1 hour. The membrane was incubated with rabbit polyclonal MVP, BCL2 antibody (1/1000 dilution) (Proteintech, Rosemont, IL), or mouse monoclonal GAPDH antibody (1/5000 dilution) (Proteintech, Rosemont, IL) overnight at 4℃. After sufficient washing, the membrane with MVP, BCL2 protein were incubated with HRP conjugated IgG anti-rabbit immunoglobulin (Proteintech, Rosemont, IL) in a blocking buffer at room temperature for 1 hour. The membrane with GAPDH protein was incubated with HRP conjugated IgG anti-mouse immunoglobulin (Proteintech, Rosemont, IL) in the same condition. The membrane was washed 3 times with PBS, and the bound antibody was detected by enhanced chemiluminescence (ECL) assay (Vazyme, Nanjing, China).

1. **Half maximal inhibitory concentration (IC50) evaluation of nanoparticle treatment**

1×10^4^ 510K cells were incubated with PBS or different NPs (CEA NPs, CEAM NPs, CEAB NPs, and CEAMB) with different concentration (0, 0.125 0.25, 0.5,1 ,2 ,4 , 8, 16, 32, 64 ug/ml NPs) for 48 hours in 96-well microtiter plates. Each concentration was replicated three times in three different wells. The culture medium was removed, and 20 μL of MTS solution was added to each well. The plate was incubated at 37℃ for another hour. Optical density (OD) was recorded at 490 nm using a microplate reader. Inhibition rates of cell proliferation in different concentration was calculated as the percentage of untreated control cells.

Inhibition rate = (OD _Untreatment_ – OD _Treatment_)/ OD _Treatment_ × 100%. Finally, the IC50 were calculated by GraphPad Prism software (Version 5).

1. **Immunohistochemistry (IHC)**

Formalin-fixed paraffin tissue sections were dewaxed with xylene for 5 min, and then rehydrated with graded ethanol. The tissue was washed with PBS 3 times for 5 min at a time. The tissue sections were then placed in a preheated 1 mm EDTA buffer (pH 8.0) for 30 minutes for antigen repair, and then cooled at room temperature. Endogenous peroxidase was inactivated and then incubated in the dark at room temperature for 15 min. A 10% FBS blocking solution was used at room temperature for 30 min and diluted rabbit polyclonal MVP, BCL2, and caspase3 antibody (1:200) (Proteintech, Rosemont, IL) were incubated overnight at 4℃. The HRP conjugate IgG anti-rabbit immunoglobulin (Proteintech, Rosemont, IL) was incubated at 37℃ for 1 h. Staining was done on the DAB (Beyotime, Shanghai, China) and visualized using the fluorescence microscope. Upon completion of the staining, the tissue sections were well rinsed in distilled water for 5 min and then counterstained in Hematoxylin solution (Sigma, St. Louis, MO) for 1 min, followed by a running tap water rinse for 3 min and then dehydrated to xylenes and sealed with neutral gum.

**Supplementary Figures Legends**


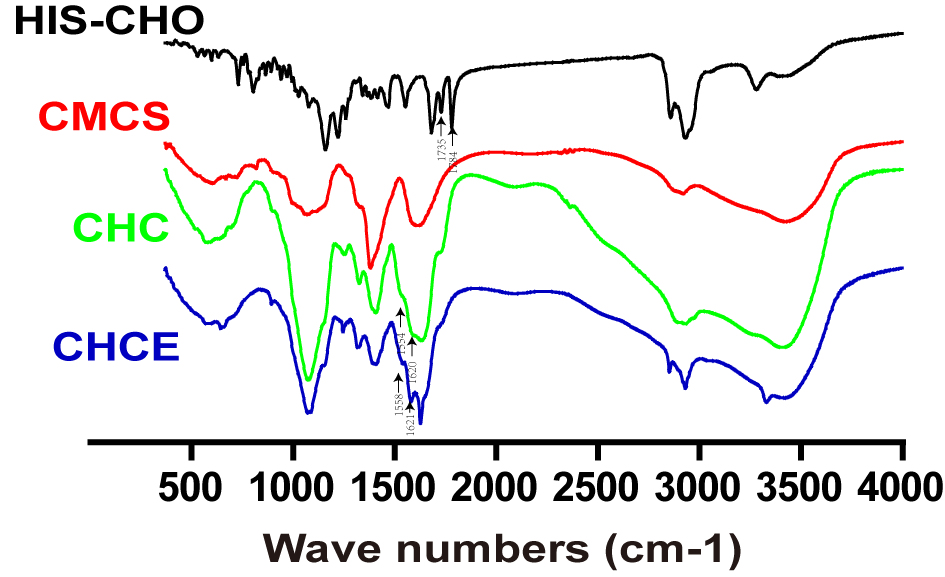


Additional file 1 Figure 1. Infrared spectra of CMCS, HIS-CHO, CHC and CHCE. Synthesis of histidine cholesteryl ester intermediate (HIS-CHO); Carboxymethyl chitosan (CMCS); Histidine cholesteryl ester modified carboxymethyl chitosan polymer compound (CHC); carboxymethyl chitosan polymer compound modified by histidine cholesteryl ester and EGFR monoclonal antibody (CHCE). By analyzing the infrared spectrum of histidine cholesteryl ester, we can know that the double peaks at 1784 cm-1 and 1735 cm-1 correspond to the symmetric stretching vibration of the carbonyl group in the ester group and the carboxyl group, respectively. This result proves that histidine and cholesterol have been an esterified reaction. In the infrared spectrum of CHC, the band at 1620 cm-1 corresponds to the stretching vibration of the carbonyl group of amide I, and the amide II band with weaker absorption at 1554 cm-1 indicates the acylation of histidine cholesterol ester and the carboxyl group of CMCS. The results indicated that the amino group of histidine cholesteryl ester was acylated with the carboxyl group of carboxymethyl chitosan to synthesize histidine cholesteryl ester modified carboxymethyl chitosan (CHC). The EGFR monoclonal antibody reacts with the amino group of CHC to generate histidine cholesteryl ester and EGFR monoclonal antibody-modified carboxymethyl chitosan (CHCE). In the infrared spectrum of CHCE, 1621 cm-1 corresponds to the stretching vibration of the carbonyl group of amide I, and the more obvious characteristic absorption peak of amide II appears at 1558 cm-1, indicating the success of the acylation reaction.


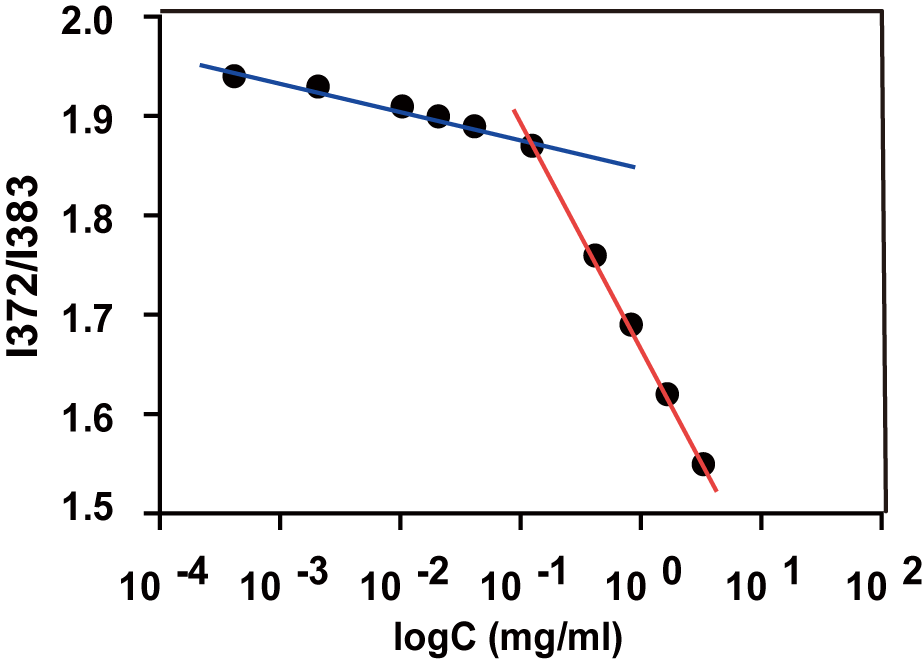


Additional file 1 Figure 2. Critical Micelle Concentration of CHCE. The intersection of the two straight lines is the critical micelle concentration of CHCE


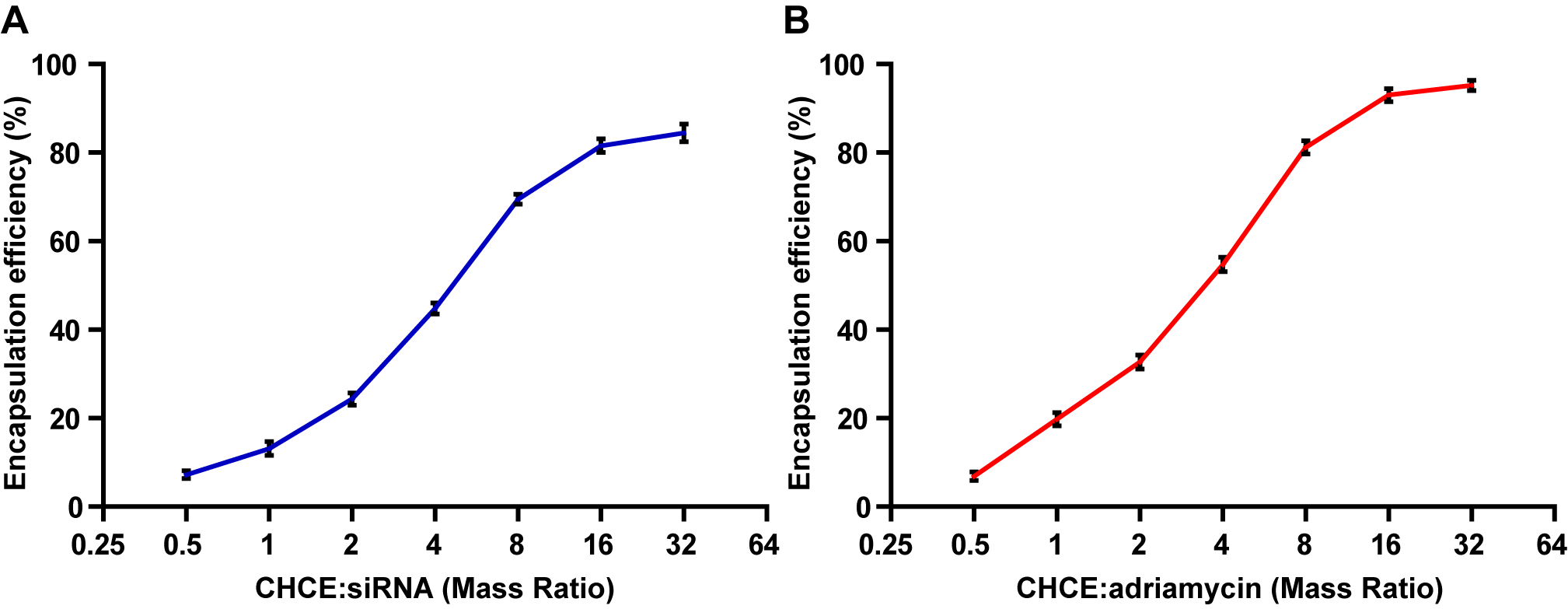


Additional file 1 Figure 3. CHCE mixed with siRNA or Adriamycin at the indicated mass ratio shown in the figure. The concentration of siRNA (A) and Adriamycin (B) was detected at a wavelength of 260 nm or 480 nm, respectively, with spectrophotometry.


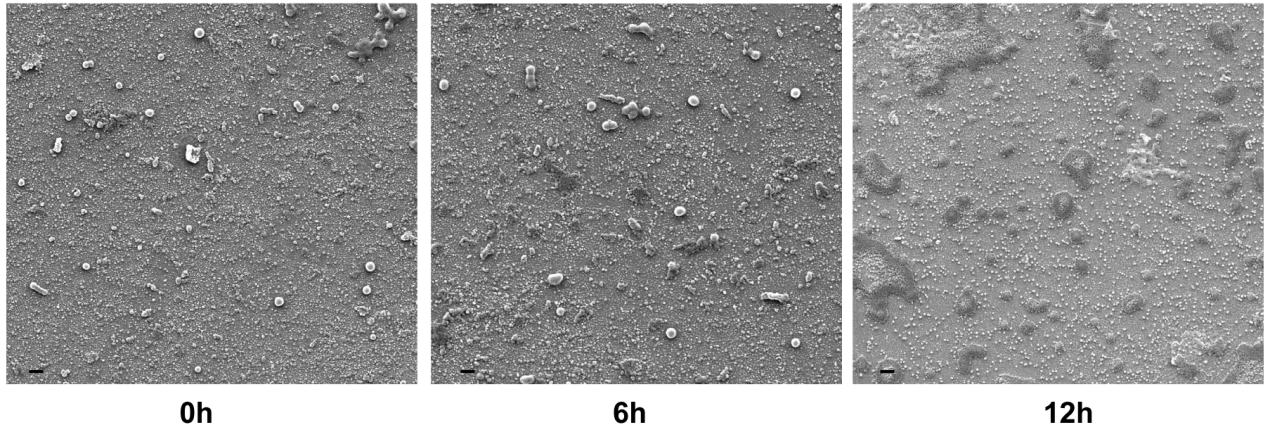


Additional file 1 Figure 4. CEAMB NPs were incubated in 50% serum at 0 h, 6 h, and 12 h at 37℃respectively. And the particle size distribution and morphology were observed under SEM. Scale bar, 200 nm.


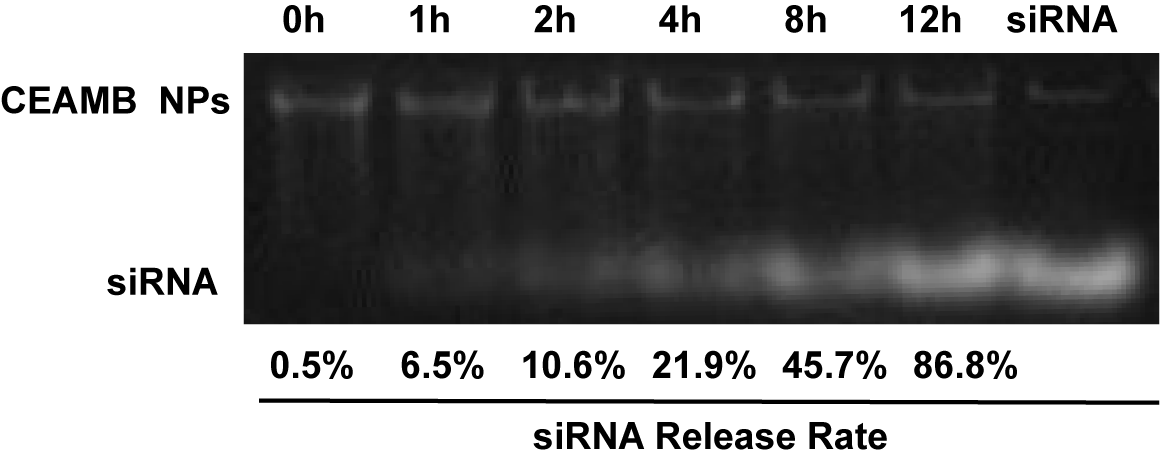


Additional file 1 Figure5. The stability of CEAMB NPs in 50% serum. CEAMB NPs were incubated with 50% serum at 37℃ at the indicated time. The gel block test was then used to detect siRNA dissociation from CEAMB NPs. In this assay, siRNA was labeled with nucleic acid dye （Ultra GelRed，10,000 ×， Nanjing V azyme Biotech Co., Ltd）, and the 1% agarose gel was used. A pure siRNA indicated the exact size of siRNA (lane 1 from the right).
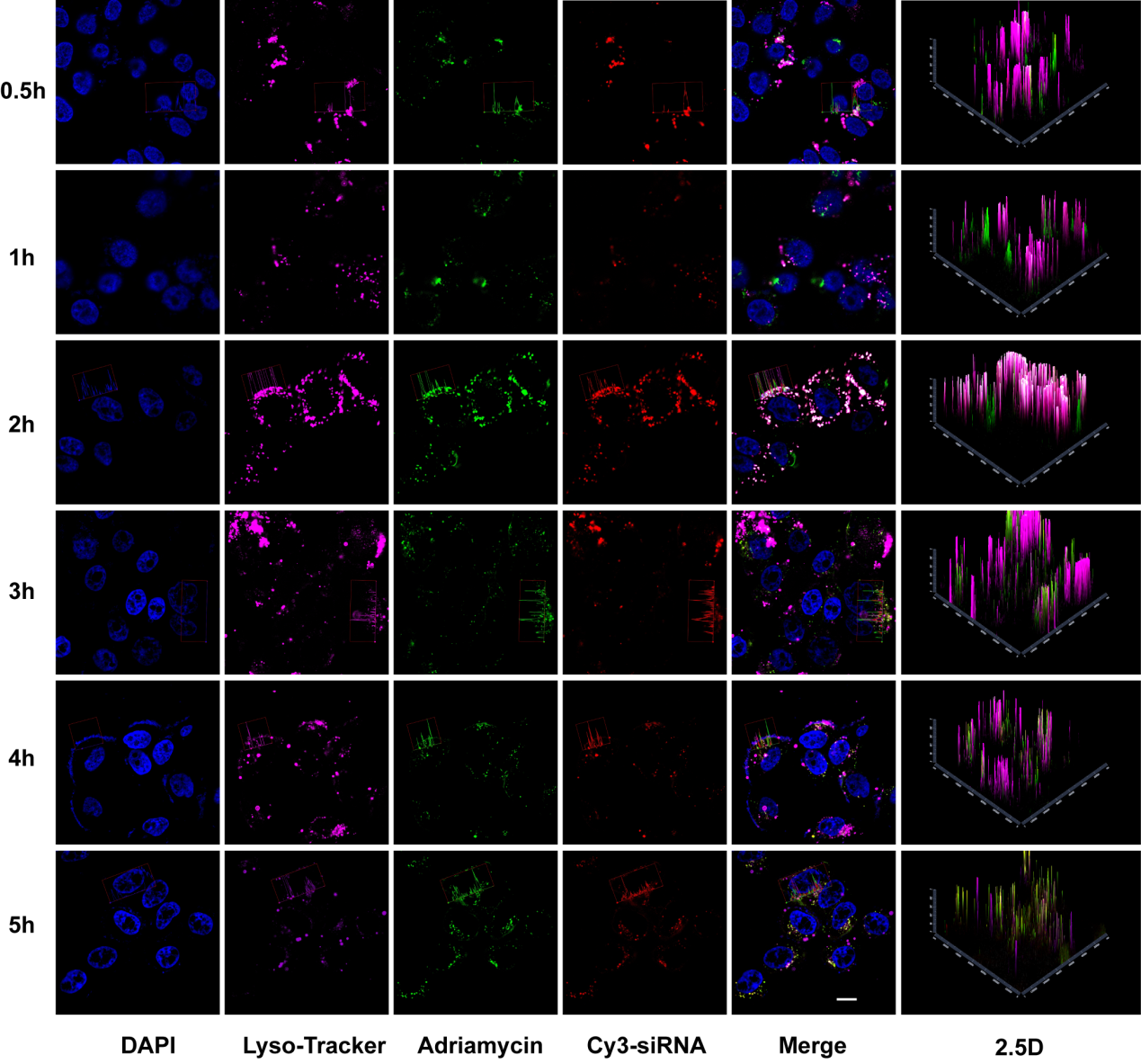


Additional file 1 Figure 6. Detection of the lysosomal escape of CEAMB NPs using Confocal Laser Scanning Microscopy. The images in this figure show the separate channels of the lysosomal with Adriamycin, or Cy3-siRNA. Conditions are the same as those in Figure 6 of the main text, and are the images of each scanned channel in Figure 6. The DAPI is blue (excitation wavelength 364 nm), adriamycin is green (excitation wavelength 488 nm), siRNA is red (excitation wavelength 532 nm), lysosome labeled by lyso-tracker is purple (excitation wavelength 577 nm) Scale bar, 10 µm.


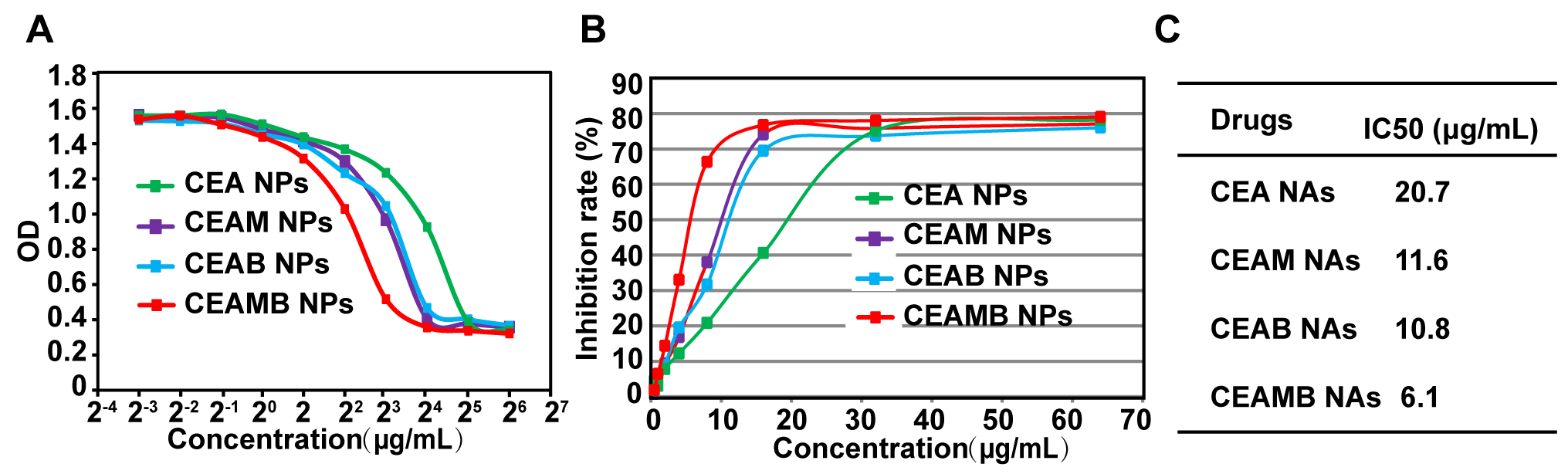


Additional file 1 Figure 7. Cytotoxicity of different NPs against 510K cells for 48 h incubation. (A) Cell viability curves of 510K cells after incubation with different drugs (mean ± s.e.m., n=3). (B) Inhibition rate of different NPs against 510K cells (mean ± s.e.m., n=3). (C) IC50 (half maximal inhibitory concentration) values of different NPs against 510K cells.


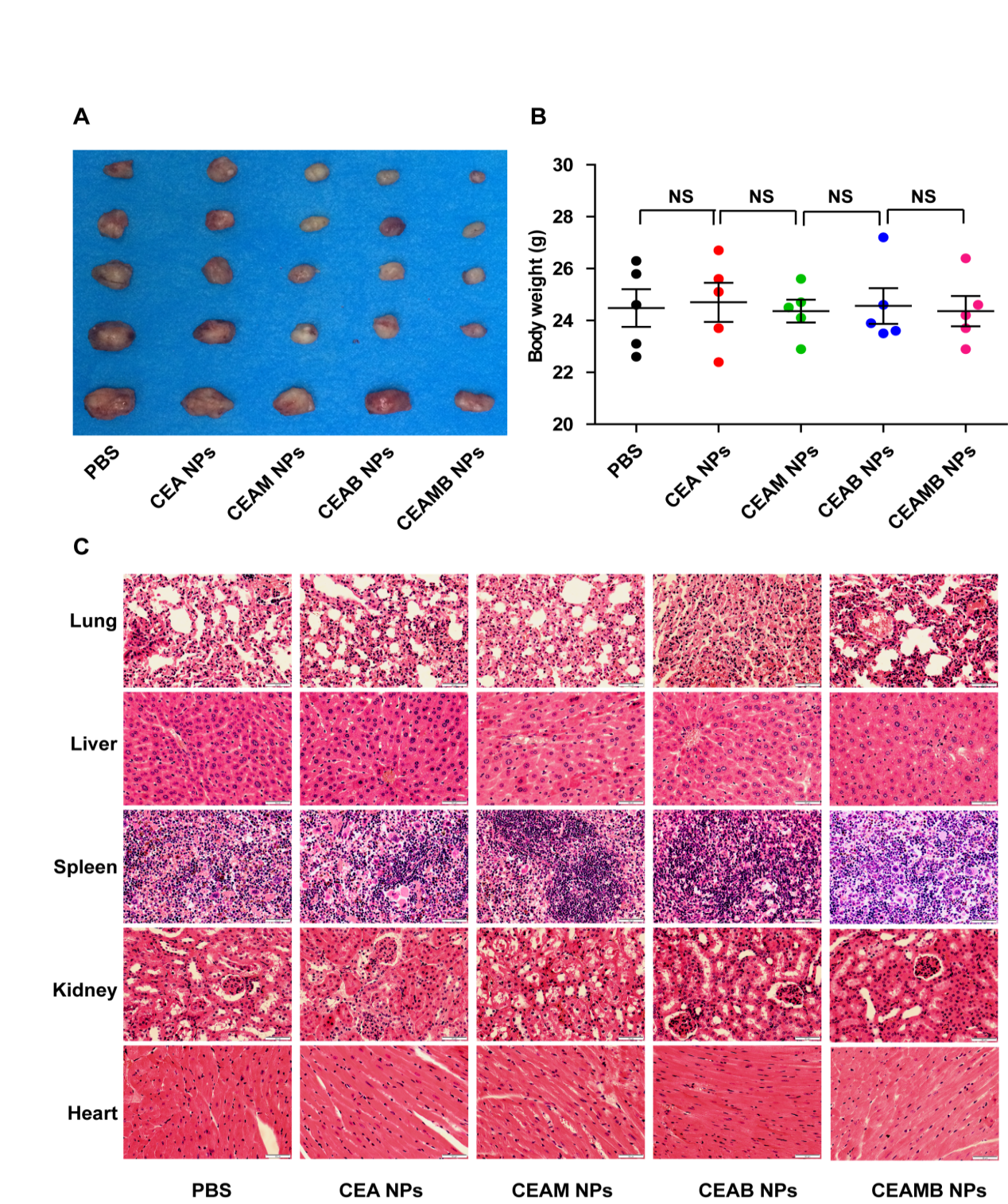


Additional file 1 Figure 8. The side effect of NPs treatment in tumor-bearing mice. K510 cells were subcutaneously injected into nude mice, and tumor engraftment was monitored. When tumor size reached 100mm3, the different NPs (CEA, CEAM, CEAB, CEAMB) and control PBS were tail vein injected for treatment. (A) At the endpoint of treatment (on day 39), the tumor of different treatment group was removed and photographed (five mice/ group). (B) the mouse body weight was measured at the endpoint, and the average weight was calculated. (mean ± s.e.m., n=5). NS, no significant difference. (C) The major organs of different treatment groups were removed and the dissected slides were used for H&E staining to detect the potential damage of NPs treatment. The typical image was shown in the figures. Scale bar = 100 µm.

Additional file 1 Table1. Primer sequences

| Primer | Primer sequence （ 5’→ 3’） |
| --- | --- |
| MVP-F | CATCACCGACCTTACGTCCA |
| MVP-R | GGGCAGGGAATTGCACTCAT |
| BCL2-F | ACAGGAGCTATACTCCAGGACA |
| BCL2-R | GATCATACCCGTCATGGGGATA |
| MRP-F | GGGAGCTTAACACCCGACTTA |
| MRP-R | GCCAAAATCACAAGGGTTAGCTT |
| P-gp-F | TTGGCTGATGTTTGTGGGAAG |
| P-gp-R | CCAAAAATGAGTAGCACGCCT |
| GST-π-F | CCCTACACCGTGGTCTATTTCC |
| GST-π-R | CAGGAGGCTTTGAGTGAGC |
| GAPDH-F | GAAGGTGAAGGTCGGAGTC |
| GAPDH-R | GAAGATGGTGATGGGATTTC |

Additional file 1 Table 2 siRNA sequences

| SiRNA | siRNA sequence（5’ → 3’） |
| --- | --- |
| MVP-nc-siRNA | sense UUCUCCGAACGUGUCACGU  antisense ACGUGACACGUUCGGAGAA |
| MVP-siRNA1 | sense AGUUCUUAUGGAAGUCAUCGA |
|  | antisense GAUGACUUCCAUAAGAACUCA |
| MVP-siRNA2 | sense CAAUGAGAGGGUACUGUUUGC |
|  | antisense CAAUGAGAGGGUACUGUUUGC |
| MVP-siRNA3 | sense UGUCUUUUCCGUAAGGAUGAC |
|  | antisense CAUCCUUACGGAAAAGACAGC |
| BCL2-nc-siRNA | sense UUCUCCGAACGUGUCACGU  antisense ACGUGACACGUUCGGAGAA |
| BCL2-siRNA1 | sense UCACUAUCUCCCGGUUAUCGU |
|  | antisense GAUAACCGGGAGAUAGUGAUG |
| BCL2-siRNA2 | sense UGUACUUCAUCACUAUCUCCC |
|  | antisense GAGAUAGUGAUGAAGUACAUC |
| BCL2-siRNA3 | sense UUGUUUCAUGGUACAUCACU G |
|  | antisense GUGAUGUACCAUGAAACAAAG |
